# Crater Lake Apoyo Revisited - Population Genetics of an Emerging Species Flock

**DOI:** 10.1371/journal.pone.0074901

**Published:** 2013-09-23

**Authors:** Matthias F. Geiger, Jeffrey K. McCrary, Ulrich K. Schliewen

**Affiliations:** 1 Bavarian State Collection of Zoology (ZSM, Zoologische Staatssammlung München), Department of Ichthyology, Munich, Germany; 2 Fundación Nicaragüense Pro-desarrollo Comunitario Integral (FUNDECI/GAIA), Estación Biológica, Laguna de Apoyo Nature Reserve, Masaya, Nicaragua; University of Massachusetts, United States of America

## Abstract

The polytypic Nicaraguan Midas cichlids ( *Amphilophus* cf. *citrinellus*) have been established as a model system for studying the mechanisms of speciation and patterns of diversification in allopatry and sympatry. The species assemblage in Crater Lake Apoyo has been accepted as a textbook example for sympatric speciation. Here, we present a first comprehensive data set of population genetic (mtDNA & AFLPs) proxies of species level differentiation for a representative set of individuals of all six endemic *Amphilophus* species occurring in Crater Lake Apoyo. AFLP genetic differentiation was partitioned into a neutral and non-neutral component based on outlier-loci detection approaches, and patterns of species divergence were explored with Bayesian clustering methods. Substantial levels of admixture between species were detected, indicating different levels of reproductive isolation between the six species. Analysis of neutral genetic variation revealed several *A* *. zaliosus* as being introgressed by an unknown contributor, hereby rendering the sympatrically evolving L. Apoyo flock polyphyletic. This is contrasted by the mtDNA analysis delivering a clear monophyly signal with Crater Lake Apoyo private haplotypes characterising all six described species, but also demonstrating different demographic histories as inferred from pairwise mismatch distributions.

## Introduction

Speciation as the cessation of gene flow between closely related populations has traditionally been classified into the three geographic modes allopatric, parapatric or sympatric, and the debate about their respective importance, prevalence and likelihood has produced hundreds of publications [1-8]. Only recently however, it has been stipulated to stop the ‘unproductive’ debates about geographic modes of speciation as well as to replace the dogmatic arguments for characterizing speciation via sexual versus natural selection by a more differentiated exploration of the relative importance of the underlying processes, and how they possibly interact [9-11]. Promising model systems for such studies are clades nested within a strong phylogenetic proximity that can be analyzed genetically, ecologically and behaviorally in as many aspects of reproductive isolation as possible.

Without confounding effects of allopatric population differentiation, crater lake cichlid species flocks, such as the Midas cichlids endemic to L. Apoyo (Nicaragua) constitute such a model to study patterns and processes of speciation due to its accessibility and feasibility to sample all species [8,12,13]. Encompassing only six described species ( *Amphilophus* *astorquii* , *A. chancho* *,* *A* *. flaveolus* , *A* *. globosus* , *A* *. supercilius* and *A* *. zaliosus* [14-16]) a comprehensive picture of the genomic diversity and divergence between them has not been presented to date. Yet, the putative monophyly of the flock has been documented based on mitochondrial DNA and nuclear markers [17-21]. However, data left room for a secondary introgression scenario from outside L. Apoyo [12,19]. Rapid speciation in sympatry leading to the formation of *A* *. zaliosus* has been hypothesized [21,22], but the likelihood of hybridization and introgression had not been investigated. Also, the contribution of the remaining L. Apoyo Midas cichlid species in this process had not been investigated before. For *A* *. zaliosus* , ecological disruptive selection has been proposed as the main factor leading to the speciation event out of the original L. Apoyo *Amphilophus* stock [21].

A common generalization from various studies and simulations is that divergence with gene flow in sympatry is easy and rapid if the naturally selected ‘magic’ traits diverge and pleiotropically lead to reproductive isolation [23-25]. Traits such as body shape or coloration under disruptive natural selection could lead to sexual isolation, if they pleiotropically affect pre-zygotic isolating mechanisms (e.g. female mate choice), and do thus promote rapid speciation. Given the very recent origin of the L. Apoyo radiation (ca. 21.000 B.P. [26]) it is not unlikely that divergently selected ‘magic’ traits integrating ecological performance and mate choice, i.e. body shape and coloration, triggered reproductive isolation.

We present results from a population genetic study of L. Apoyo’s Midas cichlids including, for the first time, samples of all six formally described species, as well as from several potential hybrid individuals. Combined findings from population genetics and phylogenetics are used to (1) infer the probability of a single colonization event postulated for the formation of a complex sympatric species flock in statu nascendi. Because this is the first study claiming to include all currently recognized L. Apoyo *Amphilophus* species level diversity, we (2) test whether there is population genetic data supporting the presence of distinct genetic clusters within L. Apoyo and whether they coincide with the described species. We (3) use genetic differentiation as measured with F-statistics, hierarchical AMOVAs and Bayesian clustering as proxies for the strength of divergence between the species and the amount of gene flow. We (4) assess signatures of introgression and hybrid speciation among sympatric L. Apoyo *Amphilophus* in the face of apparent species cohesiveness. Finally (5), the demographic history of the six species is estimated based on pairwise mismatch distributions of mtDNA haplotypes and the results are compared to those obtained from earlier studies. The work presented with its – to date – unique comprehensive taxon sampling is designed to deliver descriptive baseline data to augment the hitherto published data on parts of the L. Apoyo Midas cichlid radiation (e.g. [17-22,27]).

## Materials and Methods

The study was carried out under research permits from the Ministerio del Ambiente y los Recursos Naturales (MARENA), Nicaragua (Permit numbers: DT-MAS-MICA_C003-03-01-07 and No. 012-03007/DGAP). No endangered or protected species were involved and all procedures conformed to the animal behaviour society guidelines for the use of animals in research as well as to the legal requirements of Nicaragua and Germany. All included individuals were caught during three field seasons from January to April in 2007, December to March in 2007/2008 and April 2009 in Nicaragua. One individual of *Amphilophus* *lyonsi* (aquarium stock) was included as distantly related outgroup taxon, its adequacy supported by e.g. Concheiro et al. [28] or Říčan et al. [29], and two *Amphilophus* sp. from L. Nicaragua (Isletas) and the Rio San Juan (San Carlos) integrated as close allopatric representatives. With an estimated age of less than 23,000 years [26,30] L. Apoyo is one of the younger volcanic crater lakes in Nicaragua, situated within an almost circular caldera about 4 km west of L. Nicaragua. Its surface occupies 20.92 km^2^, its diameter measuring more than 4 km and its maximum depth 178 m [31]. Fishes were caught SCUBA diving with harpoon following preliminary field identification and anesthetized and killed using an overdose of clove oil. Each specimen was photographed to document coloration and preserved with pinned fins in 4-10% formalin. Individual whole body and tissue vouchers are stored permanently at Bavarian State Collection Munich (ZSM, Table S1). Species were identified according to the most recent species descriptions [15,16], and the key used is available from Figure S1. Specimens included 18 individuals of *Amphilophus* *astorquii* , 21 of *A* *. chancho* , 20 of *A* *. flaveolus* , 20 of *A* *. globosus* , 19 of *A* *. supercilius* and 22 of *A* *. zaliosus* . Seven individuals were included that could not unambiguously be assigned to any of the six species *a priori* - possibly due to hybrid origin.

### Phylogeny reconstruction

Genomic DNA was extracted using the Quiagen® DNeasy® 96 Tissue Kit for animal tissues according to the protocol provided by the manufacturer. Part of the mitochondrial control region was amplified using previously published primers and protocols as well as one newly designed primer: L15995 [32], H00651 [33] and H00834 (5'- ATATACACATGTCACGTAAG -3'). PCR conditions were 15 min at 95°C; 39 cycles of 95°C for 30 s, 58°C for 90 s and 72°C for 90 s, followed by 72°C for 10 min. Sequencing of the ~790 bp long fragment was done at the sequencing service of the Department of Biology of the Ludwig Maximilian University (Munich), using the Big Dye v.3.1 kit and primers L15995, H00834 and H00498 (5'- GAACCCCTTGCCCGCTAGAAAGAAT -3'). The alignment was created in ClustalX (1.81) with default settings [34]. Various sites as listed in Geiger et al. [19] were eliminated from the final alignment because they contain many single nucleotide repeats that cannot be aligned reliably. Haplotype-frequencies were calculated using Collapse 1.2 [35] with default settings, i.e. treating gaps as 5th state. A median-joining haplotype network containing all shortest phylogenetic trees (all maximum parsimony or MP trees) was constructed using NETWORK 4.5.10 following Bandelt et al. [36,37] with default settings (epsilon=0).

To study the demographic history and relative timing of the population expansion, a coalescence-based pairwise-mismatch distribution was calculated in Arlequin 3.5. The θ-estimates θ_0_ and θ_1_ are the product of 2µN_0_ and 2µN_1_ with mutation rate μ and N the effective population size at times 0 and 1. Tau (τ) is a relative measure of time since population expansion [38,39]. All mtDNA control region sequences available from Genbank on L. Apoyo *Amphilophus* individuals identified to species (Mar, 2012) were included in the pairwise mismatch analysis; their accession numbers are listed in Table S2. Plausibility of the results was checked according to Schenekar and Weiss [40], using a range of substitution rates (0.01 to 0.1 substitutions per lineage per site per MY), taking into account the variability of reported values (e.g. [41-43]).

The AFLP genotyping with 20 selective main amplifications is based on the Vos et al. [44] protocol, modified according to Herder et al. [45]. For the tree-reconstruction based on AFLP data the software package TREECON 1.3b was used [46] and the Link et al. [47] distance-measure chosen to compute a matrix based on the binary AFLP matrix.

To assess robustness of the AFLP-based phylogenetic hypothesis, and to explore alternative branching patterns, leaf-stability (LS) and lineage-movement (LM) indices for each single taxon and selected clades were calculated in Phyutility v.2.2 [48]. The LS index measures the consistency of each taxon’s position across a chosen number of bootstrap replicates. A value of 1 indicates that the individual’s position in the topology is stable and equal in all examined trees. The LM index calculates attachment frequencies of selected branches from alternative tree topologies thus identifying where a lineage is falling alternatively to its position in the tree based on the complete (non-bootstrapped) matrix [48].

To test for homoplasy-excess possibly introduced by hybrid taxa, a tree-based method as suggested by Seehausen [49] was applied. The inclusion of a hybrid taxon introduces homoplasy with clades containing the hybrid’s parental lineage due to their mosaic composition of the genome. Removal of a hybrid should decrease the amount of homoplasy and thus increase bootstrap support for clades containing hybrid parents or their descendants. Conversely, removal of non-hybrid taxa should not affect bootstrap support of other nodes.

For visualization of conflicting phylogenetic signal the Link et al. distance matrix was used to create a phylogenetic network based on the Neighbor-Net algorithm [50] as implemented in SplitsTree [51].

### Outlier locus detection

In order to discern between patterns of neutral processes and selection, a Bayesian method was applied to identify potential candidate loci under selection, implemented in BAYESCAN 1.0 using default settings [52]. Jeffreys’ scale of evidence for Bayes factors (BFs) was applied to identify candidate loci using the strictest criteria ‘decisive’, corresponding to BFs greater 100 and selection posterior probabilities above 99%. Performance of BAYESCAN was evaluated with an alternative software program to identify loci under directional selection: Dfdist (www.rubic.rdg.ac.uk/~mab/stuff/), most commonly used for AFLP markers [53].

### Inference of genetic structure

First, the Bayesian algorithm of STRUCTURE 2.2 [54,55] was used to identify the number of differentiated clusters without *a priori* group designation based on the complete AFLP matrix. The admixture-model with correlated allele-frequencies [56] was chosen, and α (admixture parameter) and λ (allelic frequencies parameter) were set to be inferred from the data. Each run consisted of a burn-in period of 50k followed by 250k iterations for posterior-probability estimates. All runs for each single K (number of populations or clusters) were replicated at least 20 times, where K ranged from 1 to 11. Runs that did not converge during the burn-in phase were identified by log. probability vs. iteration plots and removed from further analysis. Complementary, the approach of Evanno et al. [57] was applied to detect the uppermost hierarchical level of genetic structure by calculating ΔK from the STRUCTURE output ‘LnP(D)’.

After inferring the most likely number of genetic clusters the GENSBACK (gb) option in STRUCTURE [54] was used to test for immigrant ancestors. This model makes use of prior population information for each individual assisting the clustering process and calculates the probabilities that each individual was either from its predefined population (here species) or had a parent or grandparent from any other species (i.e. gb=2). Sensitivity of the data to *v* (probability of individual misclassification or mixed ancestry), was evaluated by varying the parameter MIGRPRIOR (0.1, 0.05, and 0.01), as suggested by Pritchard et al. [54]. We thus tested for immigrant ancestors from the last two generations and used for the seven potential hybrids as predefined species those with which they clustered in the NJ tree. Additionally, we performed STRUCTURE runs and principal components analysis (PCA) as graphical representation of the effect of the outlier loci on population differentiation for the AFLP matrix containing outlier loci only, and on the presumably neutral AFLP matrix without outlier loci. The structure of genetic diversity was investigated using hierarchical AMOVAs as implemented in Arlequin 3.11 for both, the mtDNA and AFLP data independently. Molecular variance was estimated among and within (1) the six species and (2) the three sampling locations. Loci identified to be under directional selection were removed from the AFLP matrix and AMOVAs as well as F-statistics recalculated as a measure of presumably neutral differentiation. Differentiation between species was estimated using F-statistics on uncorrected p-distances [58] as implemented in Arlequin 3.5, and their significance tested by permutating haplotypes among populations as well as generating bootstrap confidence intervals. For the AFLP data set, additionally pairwise *Ф*_ST_ based on standard Jaccard coefficient were generated after distance transformation (d=1-s) in FAMD [59].

### Data Accessibility

DNA sequences: GenBank accessions GU355718- GU355726; GU355729- GU355733; GU355735; GU355737; GU355739- GU355742; GU355744- GU355748; GU355764; GU355770; GU355850; GU355851; GU362707- G362709; JF784052-JF784149.

DNA aliquots: stored permanently at ZSM DNA-Bank, available upon request from dnabank@zsm.mwn.de

## Results

### Phylogenetic relationships

**Figure 1:**
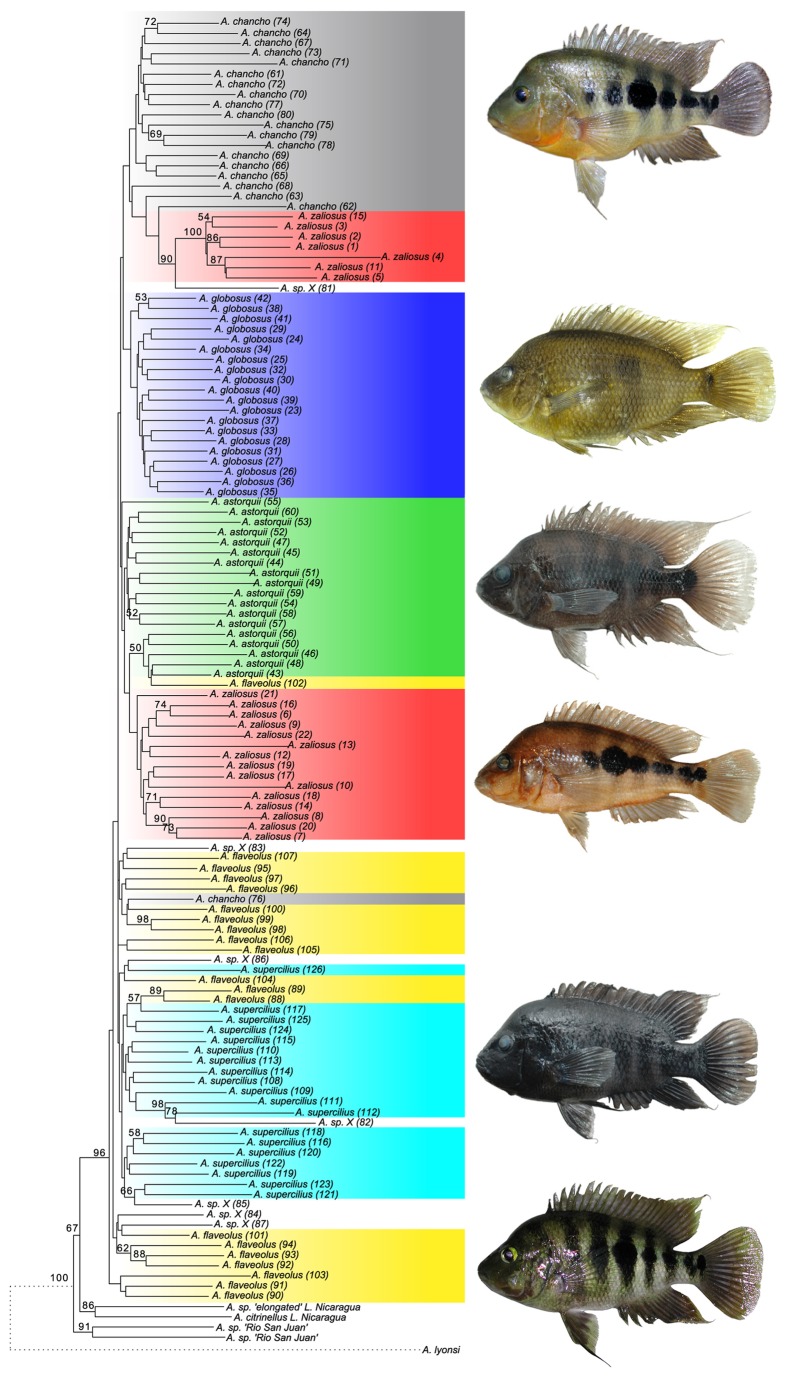
Neighbor-joining tree based on Link et al. distance measure using 2297 AFLP loci with bootstrap values ≥ 50 given above nodes.

In terms of a phylogenetic species concept the recovered NJ-phylogeny delivered different but overall low levels of monophyly support for the six L. Apoyo Midas cichlid species. The topology of the NJ-tree based on the Link et al. [47] distance measure on 2297 AFLP loci contains both taxonomically homogeneous and heterogeneous clades (Figure 1).

The single species forming a monophylum is *A* *. globosus* , whereas genotypes of all other species do not group monophyletic: *Amphilophus* *chancho* forms a cluster together with seven *A* *. zaliosus* individuals; *A* *. astorquii* falls into two main clusters, one including an *A* *. flaveolus* and being sister to the second *A* *. zaliosus* assemblage; *A* *. flaveolus* is situated basal to the Apoyo in-group and falls into several clusters with affinities to *A* *. supercilius* ; the latter species also falls into several clusters, although closely associated ones. Several individuals were not grouped according to our species identification and will be discussed later. Mean leaf-stability indices (Table S1) were similarly low for most species and individuals ( *A* *. astorquii* , m=0.55; *A* *. chancho* , m=0.56; *A* *. flaveolus* , m=0.55; *A* *. supercilius* , m=0.58; *A* *. zaliosus* , m=0.57, without the seven conspicuous *A* *. zaliosus* individuals which had elevated LS indices m=0.81), except for *A* *. globosus* (m=0.65). The included potential hybrid individuals did not show conspicuously in- or decreased LS indices.

As a consequence of the recovered topology with rather heterogeneous clades, the tree based tests for homoplasy-excess and lineage-movement where only performed for selected clades and not for species specific groups only. We conducted 36 removal experiments and excluded one at a time either each clade with bootstrap support (BS) >25%, all individuals of a single species or each of the potential hybrid individuals. Conspicuous results were the increase of BS for the monophyly of *A* *. globosus* from m=30.2% to 50% upon removal of *A* *. chancho* , and the monophyletic grouping of the otherwise non-monophyletically *A* *. zaliosus* upon removal of either *A* *. chancho* (BS=58%), *A* *. astorquii* (BS=39%) or *A* *. supercilius* (BS=29%). This hints to a possible hybridization component in the speciation event of the latter three species.

**Figure 2:**
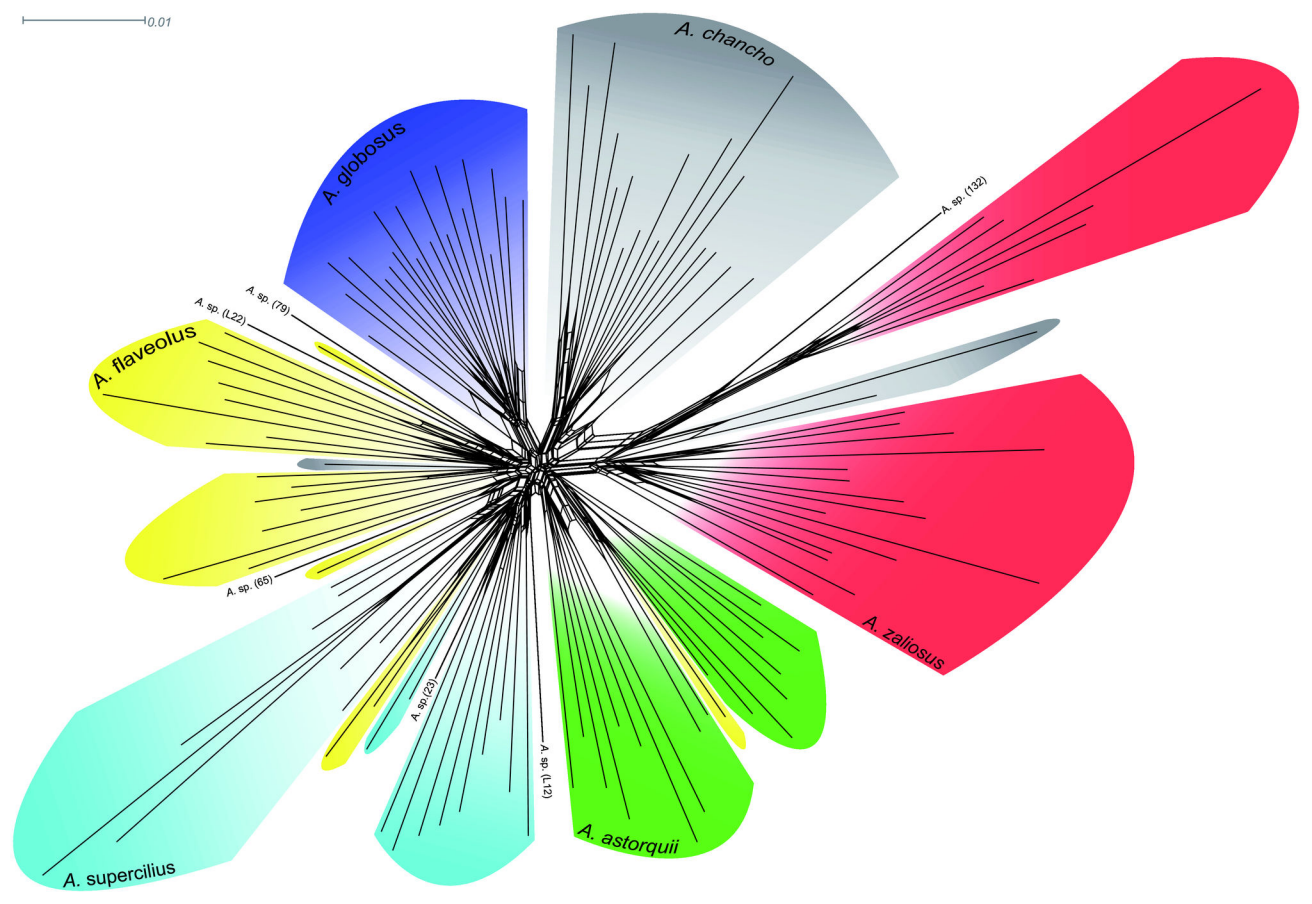
AFLP neighbor-network based on Link’s genetic distances.

The lineage-movement exploration showed that the seven conspicuous *A* *. zaliosus* individuals appeared in 38.8% of 2000 bootstrap replicates outside the whole Midas cichlid species complex as basal sister to *A* *. lyonsi* . This was only found in 4% for *A* *. zaliosus* excluding the seven conspicuous individuals and in 14% for *A* *. chancho* but not for any remaining species. The in-group neighbor-net derived from genetic distances calculated with the Link et al. algorithm [47] shows the same major groups as the NJ-tree but also indicates that there is conflicting signal at the base of the L. Apoyo radiation as indicated by numerous reticulations (Figure 2).

### Mismatch distribution & mtDNA haplotypes

**Figure 3:**
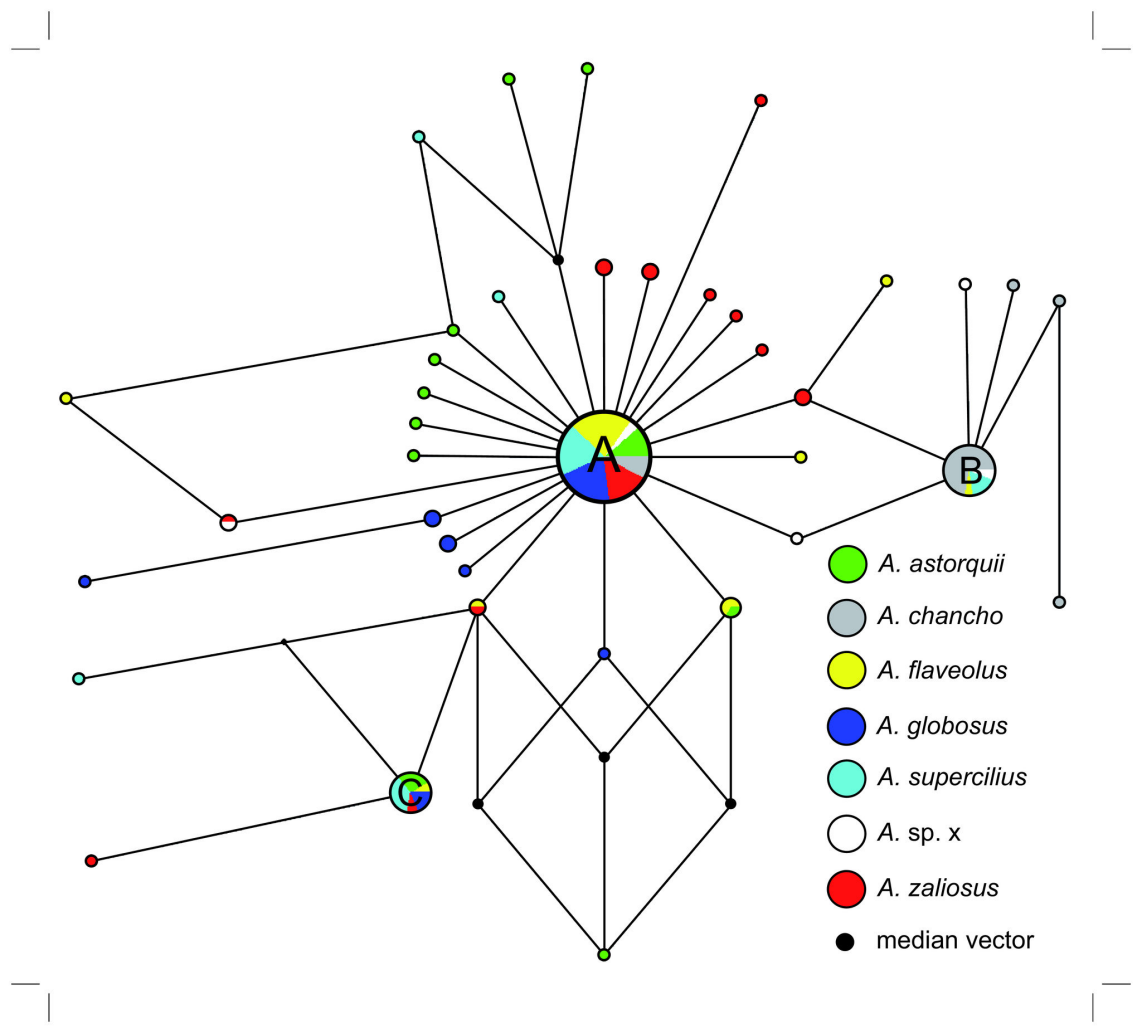
Median-joining parsimony-network based on the mitochondrial control-region, containing nine equal parsimonious trees. Circle size corresponds to sample size, branch length correlates with number of mutational steps.

**Figure 4:**
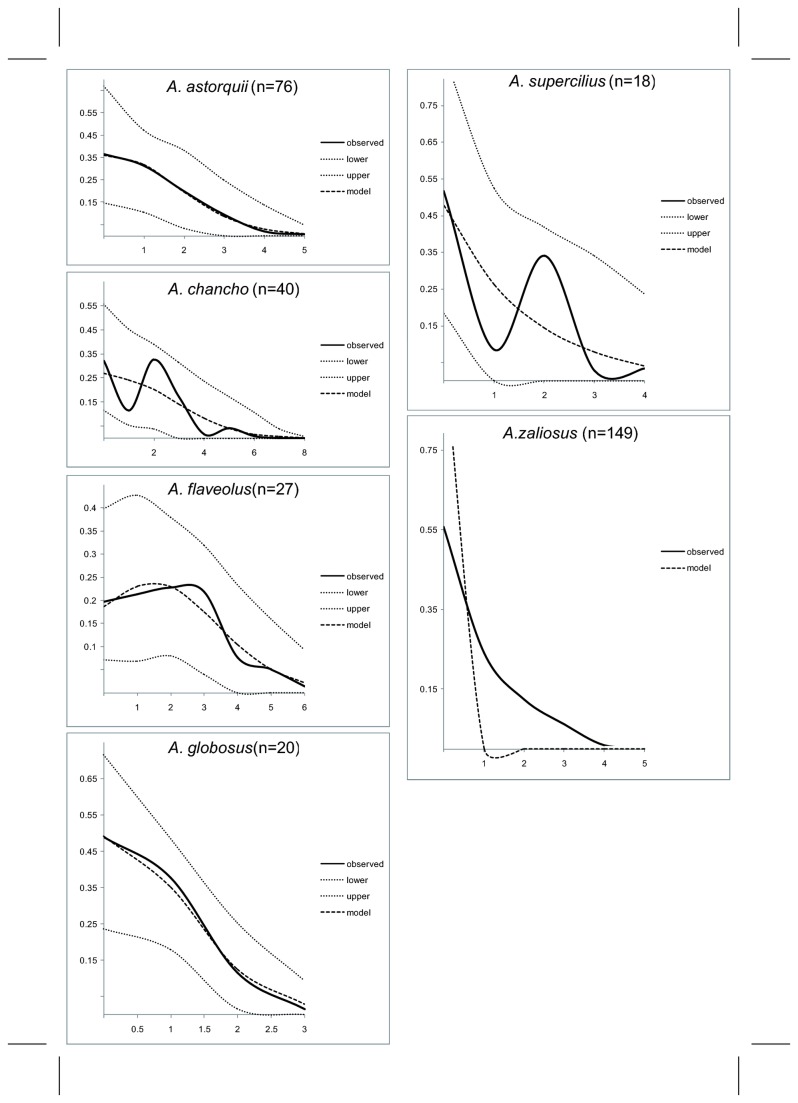
Coalescence-based mtDNA control region mismatch analysis of the six Lake Apoyo *Amphilophus* species showing different demographic histories. Expansions ca. two mutations ago occurred in *A* *. chancho* and *A* *. supercilius* and ongoing population expansions are detected in *A. astorquii* *,* *A* *. globosus* and *A* *. zaliosus* .

Among all 126 mtDNA control region sequences included, 38 different haplotypes were identified that are unique to L. Apoyo (NCBI Genbank, blastn search with default settings, Mar 2013). All six sampled species contain haplotypes that are closely (≤ 5 mutations) related to a central haplotype (“A”). Almost half of the 126 individuals (42.06%) from the six species carry the most common haplotype “A”. The relationships within the Apoyo Midas cichlids based on mtDNA are complex and all species share a variable amount of mitochondrial haplotypes (Figure 3). The coalescence based pairwise-mismatch distributions showed marked differences between the demographic histories of the six species (Figure 4). While *A* *. astorquii* , *A* *. flaveolus* , and *A* *. zaliosus* show rather smooth, steady sloped distributions as assumed for stable or expanding populations, there is clear multimodal, wave-like pattern in *A* *. chancho* and *A* *. supercilius* as would be predicted for populations that have experienced a sudden growth or decline [38] in the past. Rapid population expansion as deduced from large differences between θ_0_ and θ_1_ were observed in *A* *. globosus* and *A* *. zaliosus* only, and all observed mismatch distributions did not differ significantly from the distributions expected under population expansion, except in *A* *. zaliosus* (Table 1). Using the mismatch calculator provided by Schenekar and Weiss [40] we tested a range of substitution rates assuming a conservative generation time of two years to explain the observed Τ-value derived from Arlequin 3.5. We found pronounced differences in the mutation rates µ (substitutions per lineage per site per MY) in the six species, assuming that all species have evolved in L. Apoyo, i.e. within the last ca. 21.000 years (Table 1). According to this, the mutation rate in the mtDNA control region is higher in *A* *. chancho* , *A* *. flaveolus* and *A* *. supercilius* (µ=0.082 to 0.091) than in *A* *. astorquii* and *A* *. globosus* (µ=0.028 to 0.045), the latter being much closer to the reported values for different fish groups (e.g. [41] µ=0.036 [42]; µ=0.02 [43]; µ=0.022 to 0.045). However, our values do well agree with the range reported for different other Midas cichlid populations (e.g. [17] µ=0.019 to 0.0986).

**Table 1:** Estimated parameters for the pairwise mismatch analysis of the mtDNA sequence variability in the control region.

	*A* *. astorquii*	*A* *. chancho*	*A* *. flaveolus*	*A* *. globosus*	*A* *. supercilius*	*A* *. zaliosus*
Number of individuals	76	40	27	20	18	149
Mean no. of differences	1,107	1,610	2,120	0,658	1,288	0,922
Τ	1,4	2,6	2,5	0,7	2,5	0
θ_0_	0,03164	0	0,00703	0	0,5625	0
θ_1_	2,947	3,062	6,210	99999	1,175	427,35
SSD	0,00024	0,03936	0,00328	0,00113	0,07397	**0,27345**
Raggedness index	0,03278	0,13579	0,02309	0,09172	0,35059	0,12026
µ (thousand years)	0.045 (21.021)	0.091 (19.305)	0.082 (20.599)	0.028 (16.891)	0.082 (20.599)	-

sig. in bold.

Relative time since population expansion tau (τ), mutation parameter theta at time 0, before, and 1, after expansion, Harpending’s Raggedness index, sum of squared differences from mismatch analyses (SSD), and minimum µ (substitutions per lineage per site per MY) for time since expansion in thousands of years (in brackets).

### Genetic structure with and without outlier loci

Between-species genetic differentiation based on the short mtDNA control region sequence data varied greatly and delivered significant F_ST_’ s in nine out of 15 species-pair comparisons (Table 2). The smallest but still significant F_ST_ was calculated between *A* *. zaliosus* and *A* *. astorquii* (0.021), the highest between *A* *. chancho* and *A* *. globosus* (0.573). Investigation of within- and between-species variation using hierarchical AMOVAs with the mtDNA haplotype data showed that most (72%) variation is attributable to within-species differentiation, whereas 28% can be explained by between-species differences. Dividing the mtDNA haplotypes into groups according to sample location did not explain any amount of genetic variation (Table 3).

**Table 2:** Pairwise F_ST_-estimates based on the mtDNA control region, minimum and maximum values in bold (below diagonal), and *p*-values assessed by 10,000 permutations.

	*A* *. zaliosus*	*A* *. globosus*	*A* *. astorquii*	*A* *. chancho*	*A* *. flaveolus*	*A* *. supercilius*
*A* *. zaliosus*	**-**	<0.001	<0.023	<0.001	0.672	0.185
*A* *. globosus*	0.032	**-**	0.058	<0.001	<0.027	<0.009
*A* *. astorquii*	**0.021**	0.028	**-**	<0.001	0.530	0.141
*A* *. chancho*	0.514	**0.573**	0.530	**-**	<0.001	<0,001
*A* *. flaveolus*	-0.009	0.037	-0.003	0.502	**-**	0.459
*A* *. supercilius*	0.015	0.061	0.029	0.422	-0.007	**-**

**Table 3:** AMOVA hierarchical genetic analysis of pairwise differences based on the mtDNA control region data.

Source of variation	*df*	Percentage of variation	
among species	5	28.23*	
within species	113	71.77*	
among locations	2	-0.59^ns^	

* significant (10,100 permutations)

ns: not significant.

Using BAYESCAN 1.0 to identify potential candidate loci under directional selection from the AFLP matrix, 49 loci (ca. 2%) were detected to be influenced by directional selection applying Jeffreys’ scale of evidence for Bayes factors with the strictest criteria ‘decisive’ (selection posterior probabilities above 99%). Within the Dfdist analysis we used two significance levels (99% or 95%) and used as baseline F_ST_ either the average or the trimmed mean F_ST_, both provided by the Ddatacal program (part of the Dfdist package) as suggested by e.g. Pérez-Figueroa et al. [53] or Caballero et al. [60]. The software detected between one (p<0.01, average F_ST_) and 19 (p<0.05, trimmed F_ST_) loci that showed higher F_ST_ values than under neutral expectations. Loci detected by Dfdist were always among the 49 hits obtained with BAYESCAN. Further analyses of the AFLP data were conducted on both, the complete matrix and on the reduced matrix (without the 49 BAYESCAN loci), which we refer to as the neutral matrix.

All pairwise F_ST_’ s obtained from the complete and the reduced, neutral AFLP matrix were significantly greater than zero and ranged between 0.052 and 0.223 based on pairwise differences and 0.096-0.353 based on the Jaccard coefficient for the complete matrix and between 0.036-0.141 (pairwise differences) and 0.067-0.219 (Jaccard coefficient, Table 4) for the neutral matrix. Both methods delivered highly correlated results (r=0.99, p<0.001) with the *Ф*_ST_’s based on the Jaccard coefficient being on average 42 ± 2.6% higher. Exclusion of the 49 loci detected to be under selection had an equal effect on both measures (r=0.98, p<0.001): F_ST_’ s decreased by 30.8 ± 6.8% and *Ф*_ST_’s decreased by 30.3 ± 6.7%. Pairwise F_ST_’ s were differently strong influenced by the exclusion of the 49 loci, and a weak correlation between F_ST_ and decrease was observed (r=0.63, p<0.05). The effect ranged from a decrease by 42.8% ( *A* *. chancho* / *A* *. zaliosus* ) to 20.9% ( *A* *. astorquii* / *A* *. flaveolus* ).

**Table 4:** Pairwise F_ST_-estimates based on the binary AFLP matrix.

	*A* *. zaliosus*	*A* *. globosus*	*A* *. astorquii*	*A* *. chancho*	*A* *. flaveolus*	*A* *. supercilius*
*A* *. zaliosus*	**-**	**0.141/0.219**	0.098/0.171	0.099/0.170	0.092/0.163	0.119/0.207
*A* *. globosus*	**0.223/0.353**	**-**	0.105/0.177	0.099/0.166	0.098/0.160	0.112/0.184
*A* *. astorquii*	0.134/0.233	0.142/0.238	**-**	0.078/0.141	0.049/0.092	0.064/0.118
*A* *. chancho*	0.173/0.293	0.148/0.252	0.101/0.182	**-**	0.071/0.129	0.085/0.152
*A* *. flaveolus*	0.154/0.266	0.128/0.212	0.062/0.114	0.105/0.188	**-**	**0.036/0.067**
*A* *. supercilius*	0.188/0.317	0.159/0.263	0.084/0.153	0.132/0.230	**0.052/0.096**	**-**

First value corresponds to F_ST_ based on pairwise differences according to Weir & Cockerham [55], second value gives the *Ф*_ST_ based on standard Jaccard coefficient after distance transformation (d=1-s). All pairwise F_ST_ comparisons were significant based on 10,100 permutations following sequential Bonferroni correction. Lower left: comparisons based on all 2297 loci; upper right: neutral estimates based on the reduced AFLP matrix excluding the 49 loci identified by BAYESCAN. Minimum and maximum values in bold.

Strongest differentiation based on all loci was detected between *A* *. zaliosus* and *A* *. globosus* (F_ST_ 0.223, *Ф*_ST_ 0.353) and smallest, but still significant differentiation between *A* *. flaveolus* and *A* *. supercilius* (F_ST_ 0.052, *Ф*_ST_ 0.096). The discovered differentiation was not expected from the number of private alleles for each species: *A* *. zaliosus* (88), *A* *. supercilius* (53), *A* *. chancho* (48), *A* *. astorquii* (32), *A* *. flaveolus* (24) and *A* *. globosus* (21).

Results from AMOVAs with the AFLP data were similar to those obtained from mtDNA: genetic variation was partitioned as 14% among and 86% within species. Grouping individuals according to sample location delivered a small but significant amount of variation explained (1.6%, Table 5). Excluding the 49 outlier loci (2% of all loci) had a clear effect on AMOVA outcomes: among species variation decreased by one third while within species variation increased by 5%.

**Table 5:** AMOVA hierarchical genetic analysis of pairwise differences based on the complete AFLP matrix with 2297 loci and on the reduced matrix excluding the 49 loci identified as being under selection.

Source of variation		*df*	Percentage of variation	
			all loci	neutral loci only
among species		5	13.77*	9.15*
within species		113	86.23*	90.85*
among locations		2	1.58*	1.31*

* significant (10,100 permutations).

**Figure 5:**
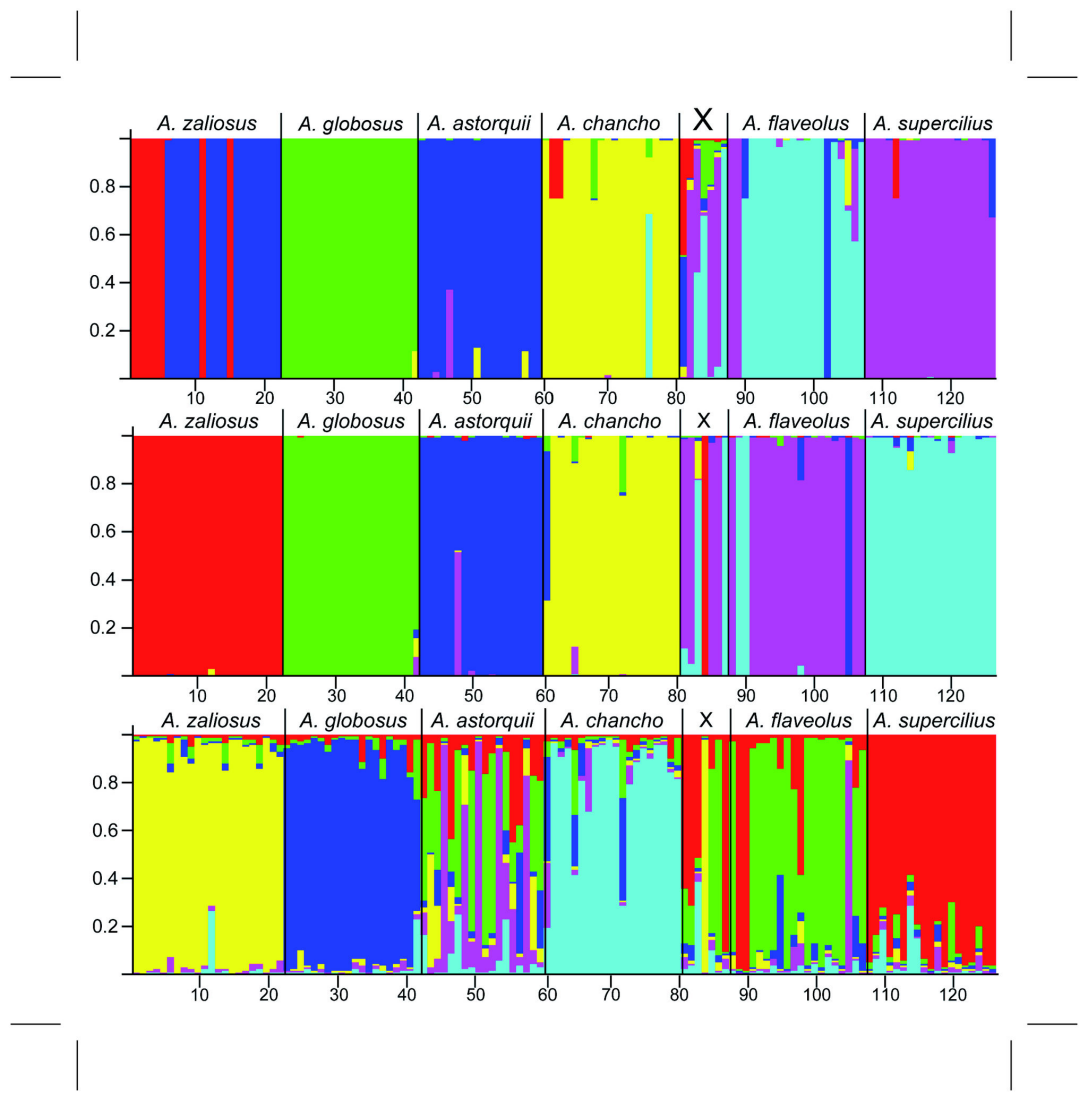
Results of STRUCTURE clustering analyses. Top: on complete AFLP matrix for K6 with group information, GENSBACK 2 and MIGPRIOR 0.05; Middle: same as top but on the 49 AFLP outlier loci only; Bottom: on the 49 outlier loci for K6 without group information. Species of sample origin given above, sample IDs of individuals given below.

Findings from the Bayesian cluster analysis with STRUCTURE v2.2 without *a priori* species designation suggest different levels of population integrity of the six species and point to the presence of hierarchical genetic structure. The Bayesian cluster method detected six clusters (K=6) to be most likely, which was also confirmed by a second peak of Evanno’s ΔK at K=6 (Figure S2), roughly corresponding to the six species. Of the 22 *A* *. zaliosus* , 15 were consistently grouped in one cluster with all *A* *. astorquii* (Figure 5, top). Repeating the STRUCTURE runs including only the latter two species shows that they do not form a genetically homogeneous group - the clustering method clearly differentiates between the two species - and shows that there are two clusters within *A* *. zaliosus* , one of which with affinities to *A. astorquii* (Figure S3), the other composed of the seven aforementioned conspicuous individuals.

Testing for misclassified or immigrant individuals in STRUCTURE with GENSBACK=2 and varying *v* (0.1, 0.05, 0.01) gave highly similar results independently of v. Mean proportions of correctly classified individuals (above 95% probability) derived from seven independent STRUCTURE runs with varying *v* were for: *A* *. globosus* 95%, *A* *. astorquii* 80.2%, *A* *. chancho* 80%, *A* *. flaveolus* 64.3% and *A* *. supercilius* 94%. As in the first analysis, 68% of the *A* *. zaliosus* where always misclassified as *A* *. astorquii* . Several individuals were constantly detected as being misclassified or having ancestry in one or more other species (Figure 5; Tables 6 & 7), and only one of the seven included potential hybrids demonstrated a clear signature of admixed origin while the remaining were mainly assigned to the species with which they clustered in the NJ tree reconstruction (Table 6).

**Table 6:** Summary table for the seven potential hybrid individuals with IDs used throughout this study.

Sample ID	81	82	83	84	85	86	87
mtDNA	*A* *. zaliosus*	A	B	A	unique	unique	C
NJ cluster	*A*.*chancho*/*zaliosus*	*A* *. supercilius*	*A* *. flaveolus*	*A* *. flaveolus*	*A* *. supercilius*	*A.flaveolus/supercilius*	*A* *. flaveolus*
STRUCTURE	97-99% *A* *. astorquii* grandparent	100% *A* *. zaliosus* grandparent	24-68% *A* *. flaveolus* & 30-66% *A* *. supercilius* grandparent	97-100% *A* *. flaveolus*	97-100% *A* *. supercilius*	99-100% *A* *. supercilius*	100% *A* *. flaveolus*
LM	*A.chancho/zaliosus* (stable)	*A* *. supercilius* (stable)	45% *A* *. flaveolus* 7% *A* *. supercilius* 2% *A* *. chancho*	40% *A* *. flaveolus* 19% *A* *. supercilius*	54% *A* *. supercilius*	28% *A* *. flaveolus* 13% *A* *. supercilius*	33% *A* *. flaveolus* 6% *A* *. astorquii*

mtDNA haplotype inferred from the parsimony network; position in the NJ tree based on Link’s distance measure; STRUCTURE results show the consensus from seven runs with varying *v* (0.01, 0.05 and 0.1) and GENSBACK=2; alternative clustering in 2000 bootstrap replicates identified by the lineage-movement procedure (LM).

**Table 7:** ‘No immigrant ancestry’ gives the probability that the ancestry of each individual is exclusively in its predefined species, following columns show the probabilities that each individual has the given amount of ancestry in the given source species.

ID	species	no immigrant ancestry	immigrant	immigrant grandparent	NJ cluster
42	*A* *. globosus*	35-83%	-	16-61% *A* *. chancho*	*A* *. globosus*
45	*A* *. astorquii*	69-96%	-	28% *A* *. supercilius*	*A* *. astorquii*
47	*A* *. astorquii*	27-66%	-	30-66% *A* *. supercilius*	*A* *. astorquii*
51	*A* *. astorquii*	40-99%	-	43-60% *A* *. chancho*	*A* *. astorquii*
58	*A* *. astorquii*	46-87%	-	37-54% *A* *. chancho*	*A* *. astorquii*
62	*A* *. chancho*	-	-	99-100% *A* *. zaliosus*	*A* *. chancho*
63	*A* *. chancho*	-	-	99-100% *A* *. zaliosus*	*A* *. chancho*
68	*A* *. chancho*	0-2%	-	98-99% *A* *. globosus*	*A* *. chancho*
76	*A* *. chancho*	-	86-95% *A* *. flaveolus*	12-15% *A* *. globosus*	*A* *. flaveolus*
88	*A* *. flaveolus*	-	100% *A* *. supercilius*	-	*A* *. supercilius*
89	*A* *. flaveolus*	-	98-100% *A* *. supercilius*	-	*A* *. supercilius*
90	*A* *. flaveolus*	16-70%	-	30-84% *A* *. astorquii*	*A* *. flaveolus*
102	*A* *. flaveolus*	-	99-100% *A* *. astorquii*	-	*A* *. astorquii*
104	*A* *. flaveolus*	30-98%	-	17-33% *A* *. supercilius*	*A* *. supercilius*
105	*A* *. flaveolus*	0-14%	-	63-87% *A* *. chancho* , 20% *A* *. supercilius*	not resolved
106	*A* *. flaveolus*	5-71%	10-75% *A* *. supercilius*	12-33% *A* *. supercilius* , 13% *A* *. astorquii*	not resolved
112	*A* *. supercilius*	0-1%	-	99-100% *A* *. zaliosus*	*A* *. supercilius*

Results show the consensus from seven runs with varying *v* (0.01, 0.05 and 0.1) and GENSBACK=2. Position in the NJ tree based on Link’s distance measure, individual’s IDs are those used throughout.

The STRUCTURE runs on the reduced AFLP matrix and on the 49 outlier loci matrix without *a priori* species assignation delivered extremely different results. No differentiated, coherent clusters were detected in the neutral AFLP matrix, irrespective of K. Only the seven *A* *. zaliosus* individuals which also clustered together in the NJ tree showed assignment probabilities around 50% to a non-Apoyo cluster (plot not shown). Quite to the contrary, including only the 49 outlier loci in the STRUCTURE runs was highly informative: 1) *K*=6 was the most likely number of genetically differentiated groups, identical to the outcome with the complete AFLP matrix; 2) all *A* *. zaliosus* individuals grouped together, while they were split based on the complete matrix; 3) a much higher percentage of individuals showed elevated admixture proportions as compared to the complete AFLP matrix and 4) only 8.9% (compared to 80.2% based on the complete matrix) of the *A* *. astorquii* individuals showed assignment probabilities above 95% to a unique cluster. Averaging over ten runs and all individuals, *A* *. astorquii* showed the following mean (+/- SD) admixture proportions: 5.97% (0.07 SD) with *A* *. chancho* , 31.5% (0.26 SD) with *A* *. flaveolus* , 6.75% (0.06 SD) with *A* *. globosus* , 14.83% (0.24 SD) with *A* *. supercilius* and 10.2% (0.05 SD) with *A* *. zaliosus* .

As with the STRUCTURE analyses on the neutral AFLP matrix and on the 49 outlier loci only, comparison of the two PCA delivered interesting insights: while there are coherent species specific clusters including all *A* *. zaliosus* individuals in a single cluster derived from the outlier loci, those clusters collapse and largely overlap except for the seven conspicuous *A* *. zaliosus* specimens that group distantly from all remaining Apoyo fishes when analysing the neutral matrix (Figure S4).

## Discussion

Our results suggest five out of the six endemic L. Apoyo Midas cichlid species to be polyphyletic with substantial levels of gene flow between them. Within *A* *. zaliosus* several individuals showed putative allochthonous phylogenetic affinities and levels of neutral differentiation not related to divergent selection within L. Apoyo indicating a possible past introgression event from outside. The demographic histories estimated from the mtDNA sequences together with the differentiation estimates based on the genome scan suggest following scenarios: primary, older splits from an ancestral form for *A* *. zaliosus* and *A* *. globosus* and secondary, more recent divergence events including a hybridization component for *A* *. astorquii* , *A* *. chancho* , *A* *. flaveolus* and *A* *. supercilius* as deduced from the homoplasy-excess tests and cluster analyses. Altogether, our findings support a sympatric *in situ* speciation scenario for all L. Apoyo Midas cichlid species.

### Outlier loci detection

Speciation is thought to begin with the rise of genomically-localised barriers to gene exchange associated with loci for local adaptation, intrinsic incompatibility or assortative mating [61]. In the early stages, gene flow will be reduced at only few loci which likely contribute to the initiation of reproductive isolation. We found that only a small proportion (ca. 2%) of the fragments show highly significant signatures of divergent selection, similarly to what had been reported for allopatric populations or ecotypes (reviewed in e.g. [62]) and also in line with a recent study on Nicaraguan Midas Cichlids including three L. Apoyo species (1.7% for L. Apoyo [20]). A comparative transcriptome study between *A* *. astorquii* and *A* *. zaliosus* identified six ESTs with signals of strong diversifying selection based on the ratio of non-synonymous to synonymous substitutions with BLAST assigned functions in biosynthetic - metabolic processes, brain development and cognition, response to hormone stimuli and in the nervous system [63]. Whether the discovered sequence differences are a product of adaptive molecular evolution or directly play a role in speciation remains yet to be tested. Resulting from the anonymity of AFLP markers used in this study, it is not possible to infer whether the detected outlier loci reside on already identified genomic regions or are linked and occur on one or few regions under selection. While the proportion of AFLP markers showing signs of selection is comparable to the aforementioned study [20], our results deviate from their finding which suggested that signatures of divergent selection in L. Apoyo are mainly driven by the divergence of *A* *. astorquii* . A similar pattern of consistently deviant allele frequencies of the detected outlier loci for *A* *. astorquii* is not present in our data. The reason for this might lie in a different sample composition with differing sample sizes when screening for outlier loci, but also in a software refinement from BAYESCAN version 1.0 to 2.01 allowing now to adjust a prior odd value for the neutral model. However, re-analysing the data with the same species sub-set as in Kautt et al. [20] and using the newer BAYESCAN 2.01 did also not result in the deviant outlier allele frequencies for *A* *. astorquii* . Kautt et al. [20] explained the pattern of deviant allele frequencies by a potential chromosomal inversion in high frequency in *A* *. astorquii* , leading to differentiation of loci within this region due to a reduction in recombination. Also, an adaptive allele on the inversion could evoke signatures of selection on multiple loci by spreading, or several adaptive alleles could reside on the same inversion, being protected from recombination and facilitating speciation [64]. As the authors state, a role of chromosomal inversions in Midas cichlid evolution is conceivable (e.g. [27,65]), but testing these genomic architectural mechanisms is only possible with markers of known position.

### Signals of ancient introgression?

The seven conspicuous *A* *. zaliosus* solely showed a strong differentiation signal in the STRUCTURE runs and PCA based on the neutral AFLP matrix. We argue that those individuals carry alleles from an unknown allopatric source, reflecting a past introgression event. They showed levels of neutral differentiation apparently not related to divergent selection within L. Apoyo. Further support for this hypothesis comes from several findings: 1) the group was characterised by four, fixed private alleles while no other species carried any fixed private alleles in our sample; 2) they clustered with the remaining *A* *. zaliosus* when considering the 49 outlier loci only, but clustered distantly from the whole Apoyo radiation based on the complete and neutral AFLP matrix (PCA & STRUCTURE); 3) the lineage-movement procedure revealed a tendency for this group to appear outside the whole Apoyo clade based on bootstrapped data. An alternative explanation to introgression from outside Apoyo is the retention of ancestral alleles that have been lost in all remaining individuals, but it is hard to explain why those alleles should have persisted just in seven individuals of a single species. It is equally unlikely that they had evolved in a subset of *A* *. zaliosus* individuals only, and perished again in the majority of sampled individuals, also rendering ‘hybrid speciation’ sensu Mallet [66] as origin of *A* *. zaliosus* implausible. We further exclude the possibility that the findings are due to technical artefacts, because the seven individuals were analysed on two different deep-well plates, contain one replicate sample, and the four fixed as well as 22 private alleles stem from different primer combinations. Interestingly, all seven individuals were collected along the western shore of L. Apoyo, their distribution thus significantly deviating from the expected distribution as judged from the remaining sampled *A* *. zaliosus* (χ^2^=12.32, df=2, p<0.001). The observed distribution might be due to philopatric behaviour with respect to breeding site, but also spatially localized genetic drift might have played a role.

The impact of the hypothesized introgression event on the speciation propensity of the L. Apoyo radiation is not fully explorable with our data, because they cannot be related to an annotated genome. The fact that the introduced alleles have apparently increased current neutral variation only does not preclude a role for them as substrate for selection directly after the putative introgression event. Hybridization and introgression can generate and increase standing genetic variation for new adaptive trait combinations suitable for exploiting resources not utilized previously [67-70]. The same mechanisms concurrently prevent the loss of genetic variation due to strong selection prevailing in sympatric speciation scenarios and may even induce speciation [66]. The product is not necessarily the formation of a hybrid species, but rather an increase in genetic variance with the introgressing form disappearing in a hybrid swarm. Theory indicates that adaptation can be more rapid if evolution acts on standing genetic variation instead of relying on the rather rare occurrence of beneficial mutations [71,72]. Our results suggest the existence of remains of an introgression event in the neutral genetic variance of *A* *. zaliosus* , thereby having increased standing genetic variation. This is also predicted by the syngameon hypothesis [49], as there was no detectable genetic structure based on the neutral AFLP matrix, indicative of a ‘common’ neutral gene pool (disregarding the allochthonous signal of the seven *A* *. zaliosus* ). Independent of that, the finding demonstrates the possibility that introgression of allochthonous alleles into seemingly fully isolated crater lake species flocks can take place. However, we find no evidence that contradicts the sympatric nature as scenario where new species emerge within a freely breeding population without geographic isolation for the L. Apoyo species flock. Theoretically possible under a variety of more or less stringent conditions (e.g. [3-6,73-76]), it is yet still considered uncommon in nature and only very few unequivocal examples are generally accepted [24,77].

### Inference of demographic history

The differences between the pairwise mismatch distributions support a scenario of stepwise species emergence – as opposed to a sudden simultaneous evolution of all six species, similar to the radiation in stages model [78], although on a much smaller scale than in the original context as model for the species rich East African cichlids [79]. However, the peculiar congruence between the strong AFLP based genetic differentiation in *A* *. globosus* and *A* *. zaliosus* and their rather steady sloped mismatch distribution which is typically for stable populations argue for a primary divergence of those two species. The clear, wavelike pattern of the pairwise mismatches in *A* *. chancho* and *A* *. supercilius* is characteristic for a sudden or exponential single growth or decline, corresponding to that event about two mutations ago. Whether this pattern was also influenced by selective sweeps in those species cannot be answered with our data. This was similarly documented for a non *A* *. zaliosus* sample from L. Apoyo of unclear species composition [18,21], where also ongoing demographic expansion was detected for *A* *. zaliosus* . In *A* *. chancho* and *A* *. supercilius* , both, the pattern of pairwise mtDNA mismatches and the AFLP based F_ST_ estimates argue for secondary divergence events after the formation of stronger isolated *A* *. globosus* & *A* *. zaliosus* populations. Notably, our and all other published demographic inferences of Midas cichlids based on the mtDNA control region (e.g. [17,18,21,80]) clearly support scenarios for *in situ* differentiation in different Nicaragua crater lakes, and never lead to pre lake-origin dates.

### Current Gene Flow

Which L. Apoyo species have been recently interconnected via gene flow? Although in the phylogenetic reconstruction *A* *. astorquii* fell into (only) two neighbouring clusters, it is, according to STRUCTURE, PCA and F_ST_ results, the species that hybridized most often. Since it is the most abundant of the six species ([81]; pers. obs.) this may also be an effect of stochasticity. It would be interesting to test whether there is a link between the species’ genomic mosaic signatures and their abundance, which could hint to a selective advantage of hybridization, but more data on biology and demography are needed to tackle this question. Admixture proportions of *A* *. astorquii* from *A* *. flaveolus* were the highest estimated, which seems to be biologically sound since they are often found in close proximity (personal observation), hereby suggesting microhabitat utilization as a factor for reproductive isolation in sympatry. But, since also other species pairs are ecologically similar and breed in direct vicinity, but are nevertheless strongly sexually isolated from each other, divergence in microhabitat use as prominent factor loses attractiveness as explanation. Interestingly, of the seven individuals classified *a priori* as potential hybrids, none grouped clearly with *A* *. astorquii* . We speculate that, although *A* *. astorquii* and *A* *. flaveolus* have apparently hybridized more frequently, resulting crosses are phenotypically less conspicuous than hybrid offspring of other crosses as e.g. between *A* *. flaveolus* and *A* *. supercilius* .

Interestingly, Kautt et al. [20] investigated the genomic signatures of divergent selection in L. Apoyo Amphilophus (excluding *A* *. globosus* , *A* *. flaveolus* and *A* *. supercilius* ), and found *A* *. astorquii* most strongly differentiated. Their pairwise F_ST_ estimates (based on approximately half the number of AFLP loci studied here) are higher but similar in scale to our estimates for *A* *. astorquii* / *A* *. zaliosus* (0.164 vs. 0.134), higher for *A* *. astorquii* / *A* *. chancho* (0.145 vs. 0.101) but substantially lower for the *A* *. chancho* / *A* *. zaliosus* (0.066 vs. 0.173) comparison. As discussed above, reasons for this might be due to different sample composition and size. Kautt et al. [20] speculate that the conspicuously long phylogenetic branches and elevated gene diversity they observed in *A* *. astorquii* hints to an increased evolutionary rate in this species. While our results also show the peculiar position of *A* *. astorquii* in the L. Apoyo radiation, we would explain their findings with the observed increased admixture proportions (Figure 5) as discussed below.

### Hybridization and Speciation

Traditionally, animal-hybrids are presumed to have reduced fitness, but recently the number of examples where hybridization apparently facilitated speciation and adaptive radiations in animals has increased [66,67,82]. Gene flow through hybridization may constitute a vector for advantageous alleles between species through backcrossing, or hybrids may show higher fitness relative to their genitors when transgressive segregation combines alleles creating novel hybrid traits [70,83-86]. It has also been hypothesized that hybridization is adaptive, leading to selection for weak discrimination between con- and heterospecifics under certain environmental conditions [87] while another extreme consequence of hybridization is despeciation [88-90]. Theoretically, reproductive isolation and functional divergence (i.e. speciation) are possible despite substantial levels of gene flow [4,91-94], a likely scenario also for L. Apoyo Midas cichlids.

It has repeatedly been put forward that hybridization is particularly advantageous where new ecological niches are created by changing or newly invaded environments (e.g. [49,67,86]). The colonization of L. Apoyo likely constituted such a situation, offering apart from reduced competition at least two novel dimensions, i.e. depth and higher water transparency, since the most likely source for the seeding population was L. Nicaragua (e.g. [17-19,21]) with mean depth ca. 13m and 0.25-0.35m Secchi disk transparency, contrasting L. Apoyo with mean depth ca. 142m and 3.5-9.5m Secchi disk transparency [95]. We know from lab experiments and field observations that ‘gold’ coloration of individual Midas cichlids affects mate choice (e.g. [96-99]), demonstrating that visual cues play a role during pair formation in Midas cichlids. An increase in transparency might thus have allowed for changes in mate choice patterns on visual cues. Water turbidity affects sexual selection by impairing the possibility for visually based mate choice [88,100,101] and also the reverse ‘speciation through sensory drive’ has been documented in Lake Victoria cichlids [102] underpinning the potential of this mechanism. Thus the conventional argument that ecological selection mediated by competition for resources among ecotypes is the primary initial driver of sympatric speciation [12,78] might also be challenged by the L. Apoyo *Amphilophus* case, where disruptive sexual selection on coloration may be a primary driver of initial reproductive isolation as recently suggested for two other prime examples for sympatric speciation [77].

## Conclusions

This is the first study based on a taxonomic comprehensive data set of population genetic proxies of species level differentiation for a representative set of individuals of all six endemic *Amphilophus* species in L. Apoyo. In summary, we find no strong indication that any of the six species has originated from ‘homoploid hybrid speciation’ sensu Mallet [66], i.e. that hybridization played a primary role in the origin of one of the six species. If so we would expect to find a similar constant signature of introgression in any of the six species as has been described for the seven conspicuous *A* *. zaliosus* individuals above. However, we demonstrate the existence of various levels of gene flow between species pairs, underlining their incipient status and a potentially non-detrimental or even beneficial role of hybridization. The observed pronounced overall genetic differentiation of *A* *. globosus* and *A* *. zaliosus* supports a scenario of primary divergence in those species as opposed to a secondary divergence including a hybridization component at least for *A* *. astorquii* , and likely also for *A* *. chancho* , *A* *. flaveolus* and *A* *. supercilius* as deduced from the homoplasy-excess tests and cluster analyses. Our comprehensive dataset supports the hypothesis of primary divergence between a limnetic and benthic habitat as suggested before for Midas cichlids from L. Apoyo [18,20] and other fish groups. In order to test the validity of the proposed secondary, more recent divergence events for *A* *. chancho* and *A* *. supercilius* several approaches seem promising. A detailed comparison of the ecology of the six species could for example show whether there might be a relationship between the sudden population growth of the latter species and their ecology. In comparison to other crater lake cichlid assemblages it could be tested whether this is a general pattern after the repeatedly demonstrated primary split along the benthic – limnetic axis. In the near future, making use of reference cichlid genomes and mapped markers to examine in detail the genome-wide divergence patterns in Midas cichlids will replace the anonymity of AFLP markers. This will allow for quantification of genomic regions under selection, but also answer questions about the underlying functional genetic basis that triggers speciation (e.g. [103-105]). For this, Nicaraguan Midas cichlids are an excellent model system, offering parallel natural experiments in independently colonized crater lakes in a well documented geological context. Given the very recent origin of the different crater lake assemblages in Nicaragua, it is likely possible to observe evolution in action and to identify not what has shaped this diversity, but what is creating diversity.

## Supporting Information

Figure S1 **Key to the six *Amphilophus* species endemic in Lake Apoyo, Nicaragua.**
(PDF)

Figure S2 **Estimation criteria for the number of genetic clusters in the AFLP data set.** Above: Mean LnP(D) with SD from 20 replicates for each K, calculated without ‘locprior’ model (STRUCTURE v2.2). Below: Evanno’s model choice criterion ‘ΔK’ for the uppermost level of genetic structure.
(PDF)

Figure S3 **Results of STRUCTURE clustering analysis with *A* *. zaliosus* and *A* *. astorquii* only for K3 using STRUCTURE v2.2 without group information.** Species of sample origin given above.
(PDF)

Figure S4 **Plot of 1st and 2nd principal component scores based on the complete AFLP matrix (A), the 49 outlier loci only (B) and the neutral AFLP matrix (C).** ID numbers are given for the potential hybrid individuals. Variance explained by 1st and 2nd PC for A) 7 & 4%, B) 18 & 10% and for C) 7 & 4%, respectively.
(PDF)

Table S1 **Sample list with individual’s ID used throughout, voucher collection number, isolate number, taxon information, sample location, leaf stability index (LS) and GenBank accession numbers.**
(DOC)

Table S2 **Genbank accession numbers of mtDNA control region sequences included in the pairwise mismatch analysis.**
(DOC)
