# Supplementary material for: Crater Lake Apoyo Revisited - Population Genetics of an Emerging Species Flock
Source: PLoS One. 2013 Sep 23;8(9):e74901. doi: 10.1371/journal.pone.0074901 (PMC3781112; doi:10.1371/journal.pone.0074901)
Supplement: Figure S1 — Key to the six Amphilophus species endemic in Lake Apoyo, Nicaragua. (PDF) [file pone.0074901.s001.pdf]

Figure S1 **Key to the six *Amphilophus* species endemic in Lake Apoyo, Nicaragua.**

1a. Shallow bodied & elongated (posterior insertion of dorsal fin to anterior insertion of anal fin is less than 34% SL or anterior insertion of dorsal fin to insertion of pelvic fin less than 42% SL) => *A. zaliosus*, black breeding coloration.

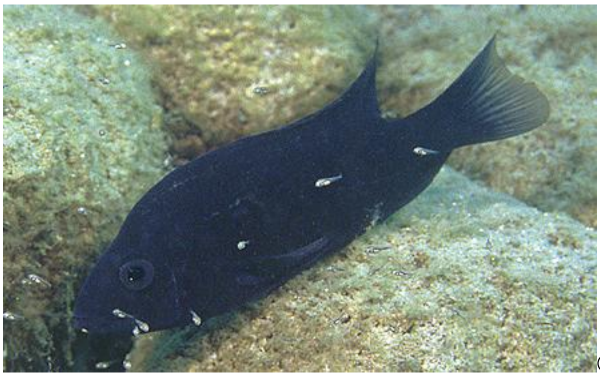
© Ad Konings

1b. High bodied (posterior insertion of dorsal fin to anterior insertion of anal fin is greater than 34% SL or anterior insertion of dorsal fin to insertion of pelvic fin greater than 42% SL) => 2

2a. Six to seven dark vertical bands absent or faint => 3
2b. Six to seven dark vertical bands present => 5

3a. Light yellow-creamy ground coloration, dark central and caudal blotch present => *A. globosus*, yellow breeding coloration.

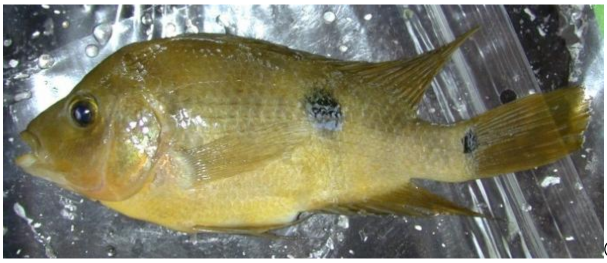
© Ad Konings

3b. Dark violet-greyish or black ground coloration => 4
4a. Caudal fin oval shaped with two outermost caudal fin rays thickened and not reaching posterior caudal fin margin => *A. supercilius*, black breeding coloration.

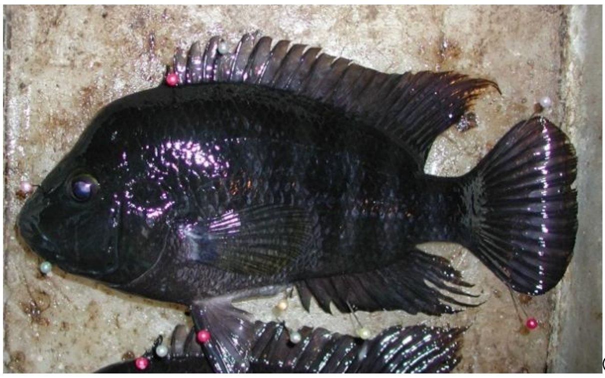© M. Geiger

4b. Caudal fin subtruncate-triangular => *A. astorquii*, black breeding coloration.

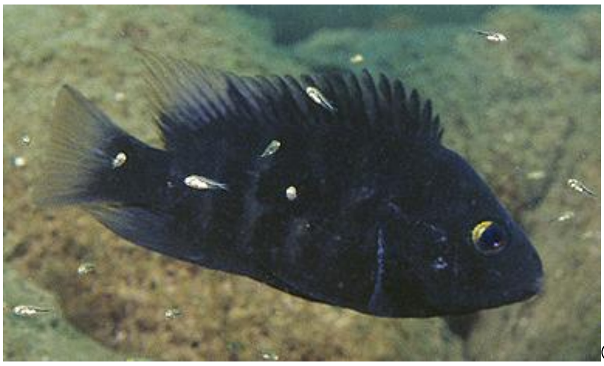© Ad Konings

5a. Yellow ground coloration, gular and breast light yellow or orange => *A. chancho*, yellow breeding coloration.

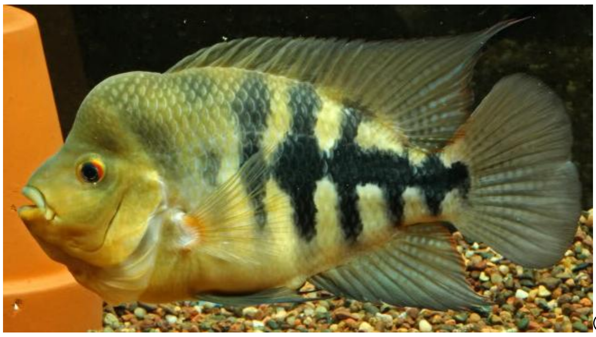© G. Markadakis

5b. Greenish ground coloration, head green => *A. flaveolus*, green breeding coloration.

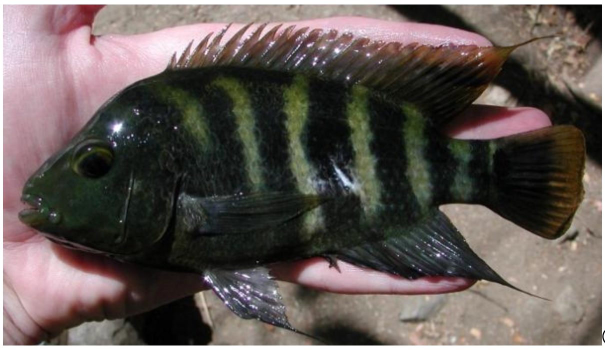© M. Geiger
