# Supplementary figures and images for: Crater Lake Apoyo Revisited - Population Genetics of an Emerging Species Flock
Source: PLoS One. 2013 Sep 23;8(9):e74901. doi: 10.1371/journal.pone.0074901 (PMC3781112; doi:10.1371/journal.pone.0074901)

K (number of populations)

2

4

6

8

10

-43000

-45000

-47000

-49000

-51000

-53000

LnP(D)

1200

12

10

8

6

4

2

$\Delta K$

1

2

3

4

5

6

7

8

9

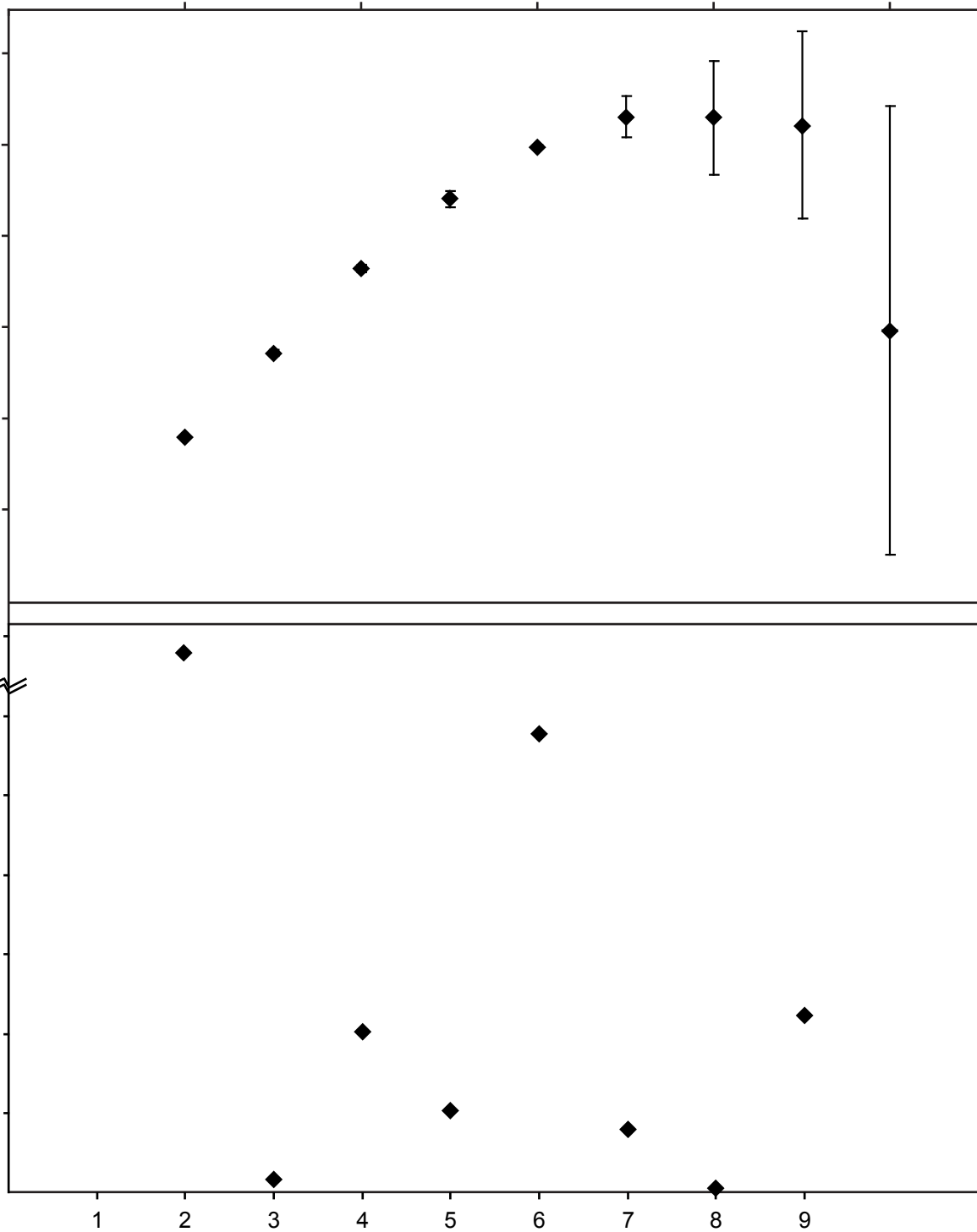

Supplement: Figure S2 — Estimation criteria for the number of genetic clusters in the AFLP data set. Above: Mean LnP(D) with SD from 20 replicates for each K, calculated without ‘locprior’ model (STRUCTURE v2.2). Below: Evanno’s model choice criterion ‘ΔK’ for the uppermost level of genetic structure. (PDF) [file pone.0074901.s002.pdf]

*A. zaliosus*

*A. astorquii*

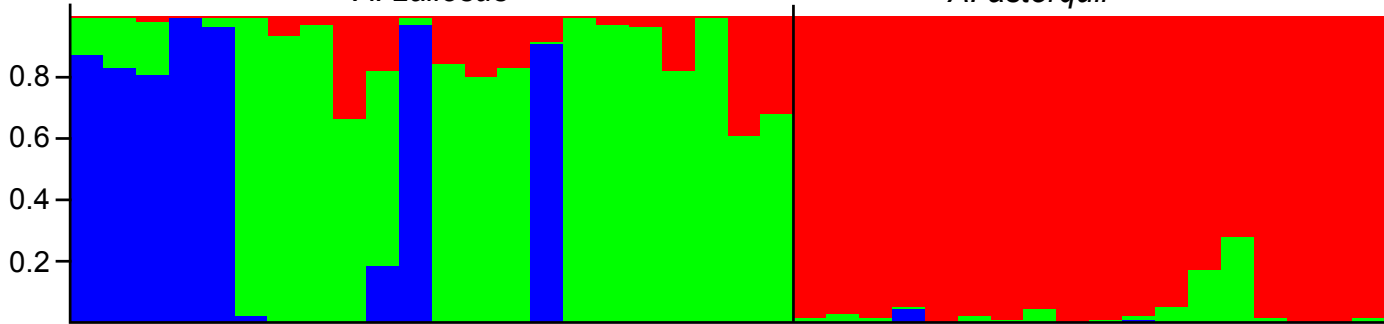

Supplement: Figure S3 — Results of STRUCTURE clustering analysis with A . zaliosus and A . astorquii only for K3 using STRUCTURE v2.2 without group information. Species of sample origin given above. (PDF) [file pone.0074901.s003.pdf]

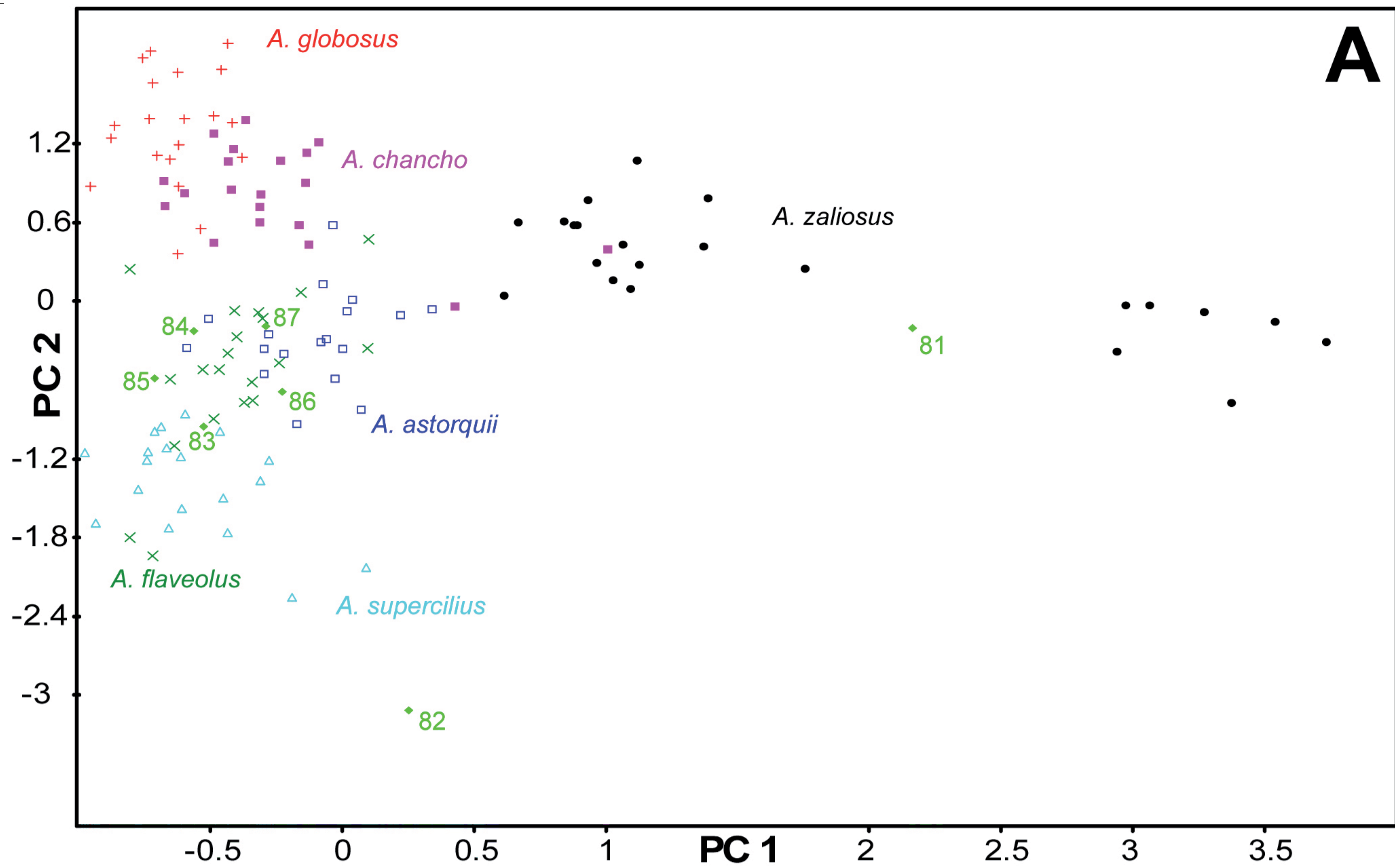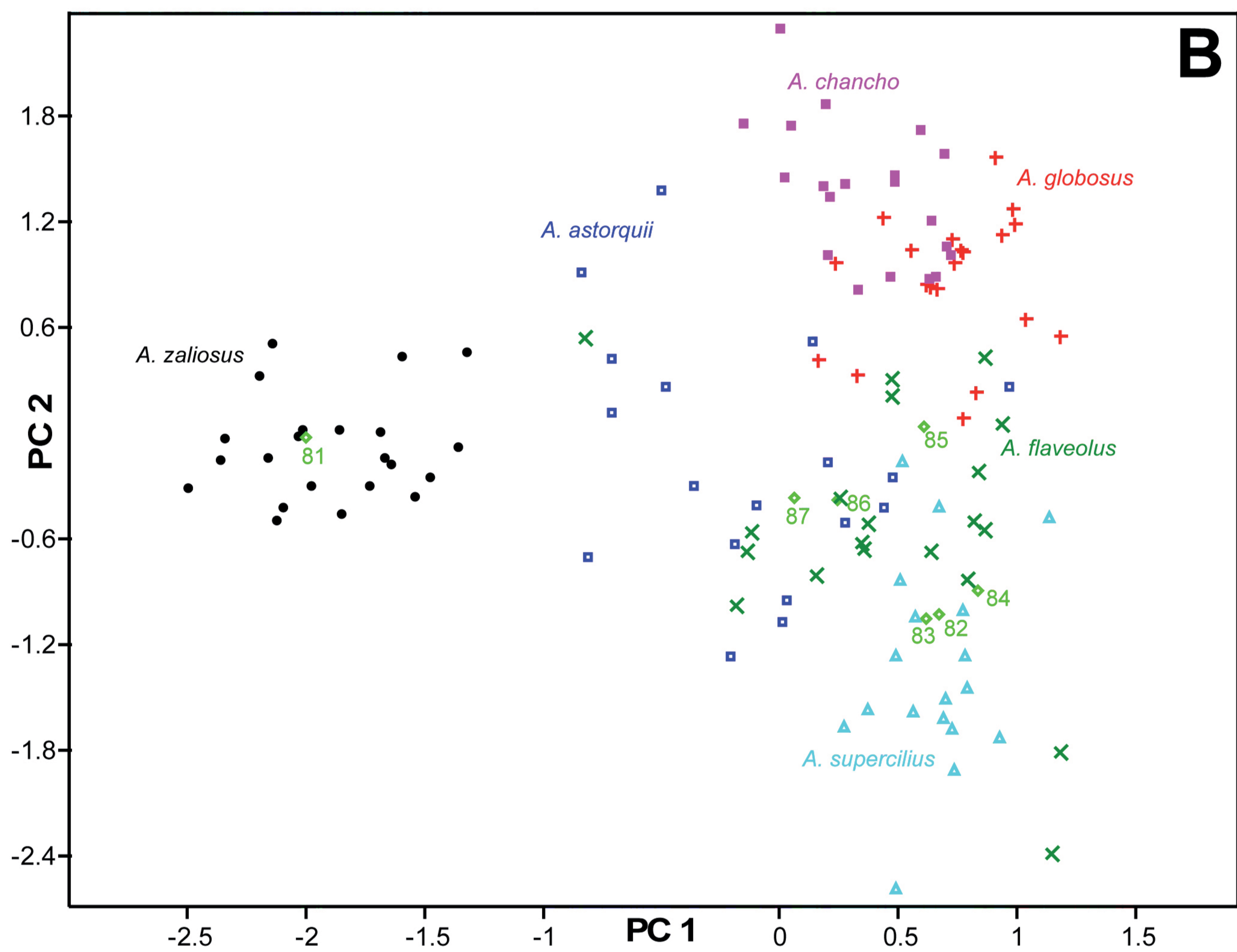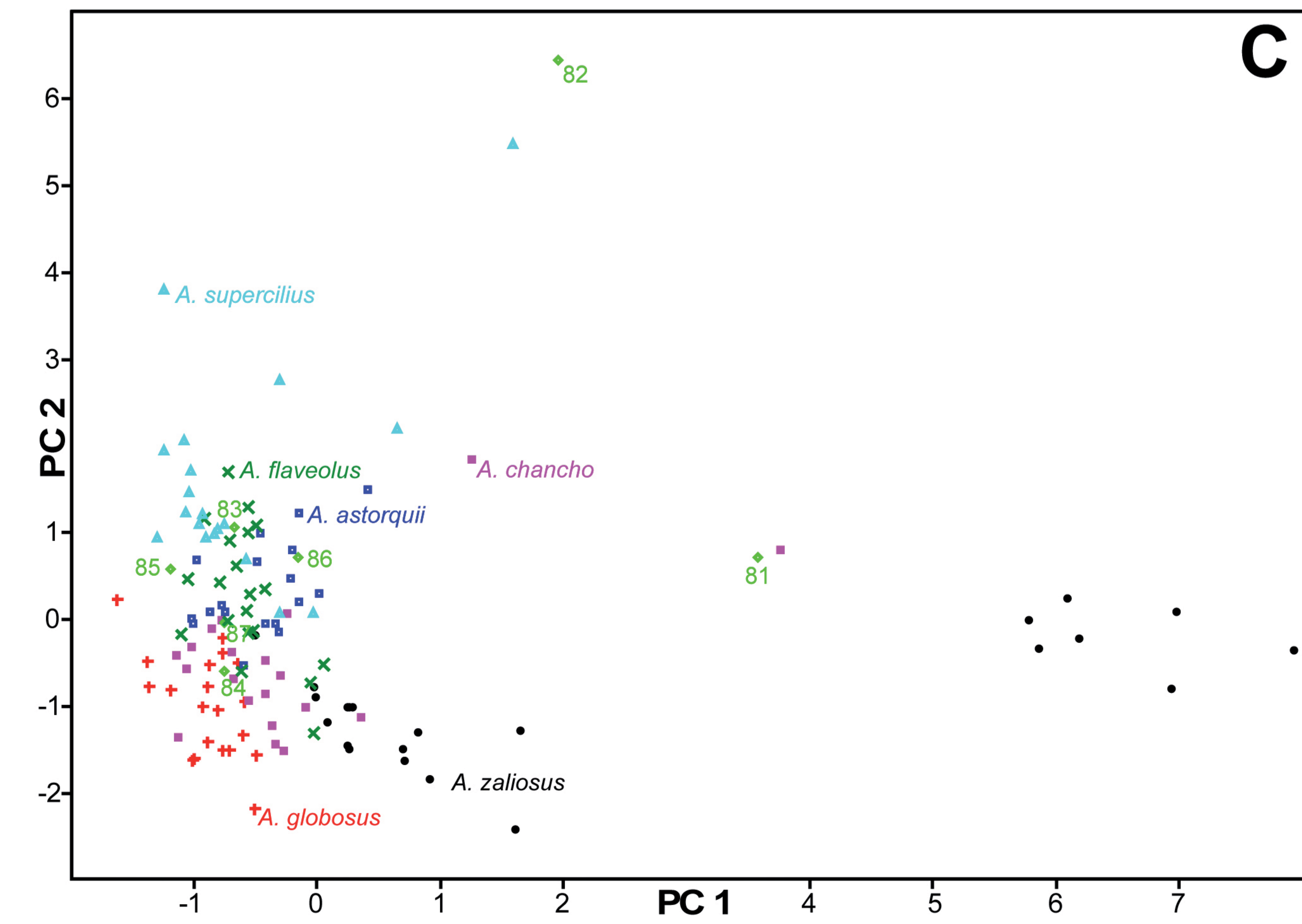

Supplement: Figure S4 — Plot of 1st and 2nd principal component scores based on the complete AFLP matrix (A), the 49 outlier loci only (B) and the neutral AFLP matrix (C). ID numbers are given for the potential hybrid individuals. Variance explained by 1st and 2nd PC for A) 7 & 4%, B) 18 & 10% and for C) 7 & 4%, respectively. (PDF) [file pone.0074901.s004.pdf]
