# Supplementary material for: Crater Lake Apoyo Revisited - Population Genetics of an Emerging Species Flock
Source: PLoS One. 2013 Sep 23;8(9):e74901. doi: 10.1371/journal.pone.0074901 (PMC3781112; doi:10.1371/journal.pone.0074901)
Supplement: Table S1 — Sample list with individual’s ID used throughout, voucher collection number, isolate number, taxon information, sample location, leaf stability index (LS) and GenBank accession numbers. (DOC) [file pone.0074901.s005.doc]

Table S1 Sample list with individual’s ID used throughout, voucher collection number, isolate number, taxon information, sample location, leaf stability index (LS) and GenBank accession numbers.

| **ID** | **Cat. #** | **tag** | **taxon** | **sample location** | **LS** | **AFLP** | **mtDNA** | **Genbank** |
| --- | --- | --- | --- | --- | --- | --- | --- | --- |
| 1 | ZSM39138 | L01 | *A. zaliosus* | Lake Apoyo W shore | 0.81 | + | + | GU362707 |
| 2 | ZSM39138 | L02 | *A. zaliosus* | Lake Apoyo W shore | 0.80 | + | + | JF784125 |
| 3 | ZSM39180 | L05 | *A. zaliosus* | Lake Apoyo W shore | 0.81 | + | + | GU355735 |
| 4 | ZSM39172 | 126 | *A. zaliosus* | Lake Apoyo W shore | 0.83 | + | + | GU355747 |
| 5 | ZSM39173 | 13 | *A. zaliosus* | Lake Apoyo W shore | 0.81 | + | + | GU355741 |
| 6 | ZSM39107 | 2 | *A. zaliosus* | Lake Apoyo W shore | 0.55 | + | + | GU355732 |
| 7 | ZSM37127 | 724 | *A. zaliosus* | Lake Apoyo S shore | 0.58 | + | + | GU362708 |
| 8 | ZSM37127 | 725 | *A. zaliosus* | Lake Apoyo S shore | 0.58 | + | + | GU362709 |
| 9 | ZSM37127 | 738 | *A. zaliosus* | Lake Apoyo S shore | 0.55 | + | + | GU355740 |
| 10 | ZSM39142 | 74 | *A. zaliosus* | L. Apoyo NE shore | 0.68 | + | + | GU355745 |
| 11 | ZSM39125 | 8 | *A. zaliosus* | Lake Apoyo W shore | 0.81 | + | + | GU355746 |
| 12 | ZSM39119 | 169 | *A. zaliosus* | L. Apoyo S shore | 0.52 | + | + | GU355742 |
| 13 | ZSM39119 | 170 | *A. zaliosus* | L. Apoyo S shore | 0.77 | + | + | GU355731 |
| 14 | ZSM39141 | 27 | *A. zaliosus* | L. Apoyo W shore | 0.57 | + | + | GU355744 |
| 15 | ZSM39141 | 31 | *A. zaliosus* | Lake Apoyo W shore | 0.81 | + | + | GU355733 |
| 16 | ZSM39140 | 51 | *A. zaliosus* | L. Apoyo W shore | 0.55 | + | + | GU355748 |
| 17 | ZSM37349 | 716 | *A. zaliosus* | Lake Apoyo NE shore | 0.54 | + | + | JF784098 |
| 18 | ZSM37350 | 717 | *A. zaliosus* | Lake Apoyo NE shore | 0.57 | + | + | JF784099 |
| 19 | ZSM37350 | 721 | *A. zaliosus* | Lake Apoyo NE shore | 0.53 | + | + | JF784102 |
| 20 | ZSM37127 | 727 | *A. zaliosus* | L. Apoyo S shore | 0.58 | + | + | GU355739 |
| 21 | ZSM39123 | 73 | *A. zaliosus* | Lake Apoyo NE shore | 0.50 | + | + | JF784114 |
| 22 | ZSM39157 | 71 | *A.* cf. *zaliosus* | L. Apoyo NE shore | 0.55 | + | + | GU355737 |
| 23 | ZSM38755 | 618 | *A. globosus* | Lake Apoyo W shore | 0.64 | + | + | JF784127 |
| 24 | ZSM38757 | 621 | *A. globosus* | Lake Apoyo W shore | 0.65 | + | + | JF784092 |
| 25 | ZSM38757 | 622 | *A. globosus* | Lake Apoyo W shore | 0.65 | + | + | JF784128 |
| 26 | PSU4745 | 626 | *A. globosus* | Lake Apoyo W shore | 0.65 | + | + | JF784131 |
| 27 | ZSM38822 | 630 | *A. globosus* | Lake Apoyo W shore | 0.66 | + | + | JF784132 |
| 28 | ZSM38758 | 631 | *A. globosus* | Lake Apoyo W shore | 0.65 | + | + | JF784133 |
| 29 | PSU4742 | 633 | *A. globosus* | Lake Apoyo W shore | 0.65 | + | + | JF784135 |
| 30 | PSU4742 | 635 | *A. globosus* | Lake Apoyo W shore | 0.65 | + | + | JF784137 |
| 31 | ZSM38758 | 636 | *A. globosus* | Lake Apoyo W shore | 0.66 | + | + | JF784138 |
| 32 | ZSM38758 | 637 | *A. globosus* | Lake Apoyo W shore | 0.65 | + | + | JF784139 |
| 33 | PSU4742 | 638 | *A. globosus* | Lake Apoyo W shore | 0.66 | + | + | JF784140 |
| 34 | PSU4742 | 639 | *A. globosus* | Lake Apoyo W shore | 0.65 | + | + | JF784141 |
| 35 | ZSM38759 | 640 | *A. globosus* | Lake Apoyo W shore | 0.66 | + | + | JF784142 |
| 36 | ZSM38759 | 641 | *A. globosus* | Lake Apoyo W shore | 0.65 | + | + | JF784143 |
| 37 | ZSM38759 | 642 | *A. globosus* | Lake Apoyo W shore | 0.65 | + | + | JF784144 |
| 38 | PSU4743 | 617 | *A. globosus* | Lake Apoyo W shore | 0.64 | + | + | JF784126 |
| 39 | PSU4744 | 623 | *A. globosus* | Lake Apoyo W shore | 0.65 | + | + | JF784129 |
| 40 | PSU4744 | 624 | *A. globosus* | Lake Apoyo W shore | 0.65 | + | + | JF784130 |
| 41 | PSU4742 | 632 | *A. globosus* | Lake Apoyo W shore | 0.63 | + | + | JF784134 |
| 42 | PSU4742 | 634 | *A. globosus* | Lake Apoyo W shore | 0.64 | + | + | JF784136 |
| 43 | ZSM39167 | 18 | *A. astorquii* | Lake Apoyo W shore | 0.55 | + | + | JF784062 |
| 44 | ZSM39159 | 20 | *A. astorquii* | Lake Apoyo W shore | 0.58 | + | + | JF784063 |
| 45 | ZSM39159 | 21 | *A. astorquii* | Lake Apoyo W shore | 0.55 | + | + | JF784064 |
| 46 | ZSM39111 | 37 | *A. astorquii* | Lake Apoyo W shore | 0.53 | + | + | JF784073 |
| 47 | ZSM39153 | 43 | *A. astorquii* | Lake Apoyo W shore | 0.58 | + | + | JF784076 |
| 48 | ZSM39153 | 48 | *A. astorquii* | Lake Apoyo W shore | 0.55 | + | + | JF784082 |
| 49 | ZSM37355 | 726 | *A. astorquii* | Lake Apoyo S shore | 0.51 | + | + | JF784105 |
| 50 | ZSM37355 | 732 | *A. astorquii* | Lake Apoyo S shore | 0.55 | + | + | JF784109 |
| 51 | ZSM37355 | 739 | *A. astorquii* | Lake Apoyo S shore | 0.50 | + | + | JF784113 |
| 52 | ZSM39166 | 84 | *A. astorquii* | Lake Apoyo W shore | 0.57 | + | + | JF784117 |
| 53 | ZSM39166 | 86 | *A. astorquii* | Lake Apoyo W shore | 0.51 | + | + | JF784119 |
| 54 | ZSM39115 | 167 | *A. astorquii* | Lake Apoyo W shore | 0.55 | + | + | JF784060 |
| 55 | ZSM39111 | 41 | *A. astorquii* | Lake Apoyo W shore | 0.56 | + | + | GU355719 |
| 56 | ZSM37354 | 720 | *A. astorquii* | Lake Apoyo NE shore | 0.55 | + | + | JF784101 |
| 57 | ZSM39110 | 35 | *A. astorquii* | Lake Apoyo W shore | 0.58 | + | + | JF784072 |
| 58 | ZSM39165 | 49 | *A. astorquii* | Lake Apoyo W shore | 0.55 | + | + | JF784083 |
| 59 | ZSM39139 | L10 | *A. astorquii* | Lake Apoyo W shore | 0.53 | + | + | JF784145 |
| 60 | ZSM39139 | L8 | *A. astorquii* | Lake Apoyo W shore | 0.55 | + | + | JF784149 |
| 61 | ZSM39132 | 127 | *A. chancho* | Lake Apoyo W shore | 0.51 | + | + | JF784054 |
| 62 | ZSM39145 | 130 | *A. chancho* | Lake Apoyo W shore | 0.72 | + | + | JF784057 |
| 63 | ZSM39169 | 30 | *A. chancho* | Lake Apoyo W shore | 0.48 | + | + | JF784070 |
| 64 | ZSM39169 | 32 | *A. chancho* | Lake Apoyo W shore | 0.56 | + | + | GU355721 |
| 65 | ZSM39109 | 34 | *A. chancho* | Lake Apoyo W shore | 0.52 | + | + | JF784071 |
| 66 | ZSM39186 | 55 | *A. chancho* | Lake Apoyo W shore | 0.52 | + | + | JF784086 |
| 67 | ZSM39130 | 57 | *A. chancho* | Lake Apoyo W shore | 0.55 | + | + | JF784087 |
| 68 | ZSM39130 | 58 | *A. chancho* | Lake Apoyo W shore | 0.46 | + | + | JF784088 |
| 69 | ZSM39176 | 60 | *A. chancho* | Lake Apoyo W shore | 0.48 | + | + | JF784091 |
| 70 | ZSM37129 | 719 | *A. chancho* | Lake Apoyo NE shore | 0.53 | + | + | JF784100 |
| 71 | ZSM37130 | 730 | *A. chancho* | Lake Apoyo S shore | 0.65 | + | + | JF784107 |
| 72 | ZSM37130 | 731 | *A. chancho* | Lake Apoyo S shore | 0.51 | + | + | JF784108 |
| 73 | ZSM37130 | 733 | *A. chancho* | Lake Apoyo S shore | 0.61 | + | + | JF784110 |
| 74 | ZSM37130 | 734 | *A. chancho* | Lake Apoyo S shore | 0.55 | + | + | GU355722 |
| 75 | ZSM37130 | 737 | *A. chancho* | Lake Apoyo S shore | 0.63 | + | + | JF784112 |
| 76 | ZSM39128 | 9 | *A. chancho* | Lake Apoyo W shore | 0.57 | + | + | JF784124 |
| 77 | ZSM39168 | 12 | *A. chancho* | Lake Apoyo W shore | 0.52 | + | + | JF784056 |
| 78 | ZSM39175 | 171 | *A. chancho* | Lake Apoyo S shore | 0.64 | + | + | JF784061 |
| 79 | ZSM39184 | 5 | *A. chancho* | Lake Apoyo W shore | 0.60 | + | + | JF784090 |
| 80 | ZSM39185 | L14 | *A. chancho* | Lake Apoyo W shore | 0.54 | + | + | JF784147 |
| 81 | ZSM39108 | 132 | *A.* sp. X | Lake Apoyo W shore | 0.70 | + | + | JF784058 |
| 82 | ZSM39164 | 54 | *A.* sp. X | Lake Apoyo W shore | 0.84 | + | + | JF784085 |
| 83 | ZSM39155 | 65 | *A.* sp. X | Lake Apoyo W shore | 0.59 | + | + | JF784093 |
| 84 | ZSM39161 | 79 | *A.* sp. X | Lake Apoyo NE shore | 0.57 | + | + | GU355730 |
| 85 | ZSM39152 | L23 | *A.* sp. X | Lake Apoyo NE shore | 0.60 | + | + | GU355718 |
| 86 | ZSM39151 | L12 | *A.* sp. X | Lake Apoyo W shore | 0.57 | + | + | JF784146 |
| 87 | ZSM39149 | L22 | *A.* sp. X | Lake Apoyo NE shore | 0.55 | + | + | JF784148 |
| 88 | ZSM39118 | 128 | *A. flaveolus* | Lake Apoyo S shore | 0.54 | + | + | GU355723 |
| 89 | ZSM39171 | 129 | *A. flaveolus* | Lake Apoyo S shore | 0.54 | + | + | JF784055 |
| 90 | ZSM39135 | 168 | *A. flaveolus* | L. Apoyo W shore | 0.49 | + | + | GU355724 |
| 91 | no voucher | 470 | *A. flaveolus* | Lake Apoyo NE shore | 0.50 | + | + | JF784078 |
| 92 | no voucher | 472 | *A. flaveolus* | Lake Apoyo NE shore | 0.52 | + | + | JF784079 |
| 93 | no voucher | 473 | *A. flaveolus* | Lake Apoyo NE shore | 0.52 | + | + | JF784080 |
| 94 | no voucher | 474 | *A. flaveolus* | Lake Apoyo NE shore | 0.51 | + | + | JF784081 |
| 95 | ZSM39116 | 68 | *A. flaveolus* | Lake Apoyo NE shore | 0.61 | + | + | JF784095 |
| 96 | ZSM39133 | 69 | *A. flaveolus* | L. Apoyo NE shore | 0.53 | + | + | GU355725 |
| 97 | ZSM39137 | 85 | *A. flaveolus* | Lake Apoyo NE shore | 0.58 | + | + | JF784118 |
| 98 | ZSM39117 | 87 | *A. flaveolus* | Lake Apoyo S shore | 0.61 | + | + | JF784120 |
| 99 | ZSM39117 | 88 | *A. flaveolus* | L. Apoyo S shore | 0.61 | + | + | GU355726 |
| 100 | ZSM39117 | 89 | *A. flaveolus* | Lake Apoyo S shore | 0.60 | + | + | JF784121 |
| 101 | ZSM39117 | 90 | *A. flaveolus* | Lake Apoyo S shore | 0.59 | + | + | JF784122 |
| 102 | ZSM39170 | 91 | *A. flaveolus* | Lake Apoyo S shore | 0.55 | + | + | JF784123 |
| 103 | ZSM39136 | 124 | *A. flaveolus* | Lake Apoyo W shore | 0.61 | + | + | JF784052 |
| 104 | ZSM39136 | 125 | *A. flaveolus* | Lake Apoyo W shore | 0.60 | + | + | JF784053 |
| 105 | ZSM39134 | 254 | *A. flaveolus* | Lake Apoyo NE shore | 0.50 | + | + | JF784068 |
| 106 | ZSM39134 | 255 | *A. flaveolus* | Lake Apoyo NE shore | 0.50 | + | + | JF784069 |
| 107 | ZSM39133 | 67 | *A. flaveolus* | Lake Apoyo NE shore | 0.55 | + | + | JF784094 |
| 108 | ZSM38751 | 164 | *A. supercilius* | Lake Apoyo W shore | 0.61 | + | + | JF784059 |
| 109 | ZSM38821 | 247 | *A. supercilius* | Lake Apoyo NE shore | 0.44 | + | + | JF784065 |
| 110 | ZSM38779 | 248 | *A. supercilius* | Lake Apoyo NE shore | 0.62 | + | + | JF784066 |
| 111 | ZSM38753 | 39 | *A. supercilius* | Lake Apoyo W shore | 0.74 | + | + | JF784074 |
| 112 | ZSM38777 | 3 | *A. supercilius* | Lake Apoyo W shore | 0.84 | + | + | JF784075 |
| 113 | ZSM38780 | 45 | *A. supercilius* | Lake Apoyo W shore | 0.62 | + | + | JF784077 |
| 114 | ZSM38751 | 50 | *A. supercilius* | Lake Apoyo W shore | 0.60 | + | + | JF784084 |
| 115 | ZSM39148 | 6 | *A. supercilius* | Lake Apoyo W shore | 0.60 | + | + | JF784096 |
| 116 | ZSM37351 | 728 | *A. supercilius* | Lake Apoyo S shore | 0.51 | + | + | JF784106 |
| 117 | ZSM37347 | 735 | *A. supercilius* | Lake Apoyo S shore | 0.56 | + | + | JF784111 |
| 118 | ZSM37351 | 736 | *A. supercilius* | Lake Apoyo S shore | 0.49 | + | + | GU355720 |
| 119 | ZSM38754 | 252 | *A. supercilius* | Lake Apoyo NE shore | 0.58 | + | + | JF784067 |
| 120 | ZSM39113 | 47 | *A. supercilius* | Lake Apoyo W shore | 0.48 | + | + | GU355729 |
| 121 | ZSM38776 | 70 | *A. supercilius* | Lake Apoyo NE shore | 0.54 | + | + | JF784097 |
| 122 | ZSM37353 | 722 | *A. supercilius* | Lake Apoyo NE shore | 0.61 | + | + | JF784103 |
| 123 | ZSM38776 | 78 | *A. supercilius* | Lake Apoyo NE shore | 0.51 | + | + | JF784115 |
| 124 | ZSM38753 | 59 | *A. supercilius* | Lake Apoyo W shore | 0.60 | + | + | JF784089 |
| 125 | ZSM38778 | 7 | *A. supercilius* | Lake Apoyo W shore | 0.53 | + | + | JF784116 |
| 126 | ZSM37348 | 723 | *A. supercilius* | Lake Apoyo S shore | 0.50 | + | + | JF784104 |
| - | ZSM39207 | 211 | *A.* cf. *citrinellus* | Lake Nicaragua, Isletas | 0.88 | + | **-** | GU355764 |
| - | ZSM39197 | 292 | *A.* sp. | Lake Nicaragua, Isletas | 0.88 | + | **-** | GU355770 |
| - | ZSM37087 | 680 | *A.* sp. RSJ | Rio San Juan near San Carlos | 0.88 | + | **-** | GU355850 |
| - | ZSM37255 | 681 | *A.* sp. RSJ | Rio San Juan near San Carlos | 0.88 | + | **-** | GU355851 |
