# Supplementary material for: Crater Lake Apoyo Revisited - Population Genetics of an Emerging Species Flock
Source: PLoS One. 2013 Sep 23;8(9):e74901. doi: 10.1371/journal.pone.0074901 (PMC3781112; doi:10.1371/journal.pone.0074901)
Supplement: Table S2 — Genbank accession numbers of mtDNA control region sequences included in the pairwise mismatch analysis. (DOC) [file pone.0074901.s006.doc]

Table S2 Genbank accession numbers of mtDNA control region sequences included in the pairwise mismatch analysis.

*Amphilophus astorquii* (n=58): HM183527.1 - HM183544.1; HM183555.1 - HM183559.1; HM183561.1; HM183569.1; HM183583.1 - HM183599.1; HM183602.1; HM183606.1; HM183607.1; HM183609.1 - HM183615.1; HM183620.1 - HM183624.1; HM183626.1

*Amphilophus chancho* (n=20): HM183545.1 - HM183551.1; HM183560.1; HM183570.1 - HM183576.1; HM183600.1; HM183601.1; HM183604.1; HM183605.1; HM183617.1

*Amphilophus flaveolus* (n=6): HM183562.1 - HM183565.1; HM183603.1; HM183625.1

*Amphilophus zaliosus* (n=125): AY567376.1 - AY567410.1; DQ229964.1 - DQ229966.1; DQ229969.1; DQ229970.1; DQ229972.1 - DQ229975.1; DQ229977.1; DQ229979.1; DQ229981.1; DQ229985.1 - DQ229989.1; DQ229992.1; DQ229993.1; DQ229996.1; DQ230007.1 - DQ230010.1; DQ230015.1; DQ230019.1 - DQ230021.1; DQ230027.1; DQ230048.1; DQ230051.1 - DQ230058.1; HM183496.1 - HM183526.1; HM183566.1 - HM183568.1; HM183577.1 - HM183579.1; HM183581.1; HM183582.1; HM183608.1; HM183618.1; HM183619.1; HM183627.1 - HM183636.1
